# Efficacy of sulfonamides targeting malic enzyme in an animal model of Chagas disease

**DOI:** 10.3389/fphar.2025.1709223

**Published:** 2025-11-28

**Authors:** Thaís Cristina Ferreira dos Santos, Ramon Borges da Silva, Irene Layane de Sousa, Fabrício Fredo Naciuk, Letícia Marchese, Amanda Gonçalves Eufrásio, Angel Eduardo Lobo-Rojas, Renan Marcel Giampauli, Valéria Barbosa de Souza, André Almeida Schenka, Marjorie Bruder, Silvana Aparecida Rocco, Artur Torres Cordeiro

**Affiliations:** 1 Brazilian Biosciences National Laboratory (LNBio), Brazilian Center for Research in Energy and Materials (CNPEM), Campinas, São Paulo, Brazil; 2 School of Pharmaceutical Sciences, Universidade Estadual de Campinas (UNICAMP), São Paulo, Brazil; 3 Department of Pharmacology, School of Medical Sciences, Universidade Estadual de Campinas (UNICAMP), São Paulo, Brazil

**Keywords:** acute phase, ADME, bioavailability, Chagas disease, malic enzyme, sulfonamides

## Abstract

**Introduction:**

Even after a century since its discovery, Chagas disease remains a major public health concern. Benznidazole and nifurtimox are the only approved treatments, but their limited efficacy and adverse effects highlight the urgent need for new therapies. In the last decade, several phenotypic screenings performed by pharmaceuticals and academic groups revealed new promising compounds. In a previous study, we identified the *T. cruzi* malic enzyme (TcME) as the molecular target for the sulfonamide TCMDC-143108, a phenotypic hit made public available through the Chagas Box collection. Indeed, we determined crystallographic structures for TcME-inhibitor complexes and synthesized new molecules with improved activity against intracellular *T. cruzi* forms.

**Aim:**

The present study aims to evaluate the efficacy of new sulfonamides derived from TCMDC-143108 in an animal model for acute Chagas disease.

**Methods:**

The new sulfonamides were evaluated for TcME inhibition and activity against *T. cruzi* Dm28c strain, infecting h9c2 cardiomyoblast. Active compounds progressed to ADME *in vitro* assays. Promising hits were assessed for oral bioavailability. *In vivo* efficacy was evaluated in infected balb/c mice by direct parasitemia, parasite tissue burden (heart, colon, and spleen), and histopathological examination.

**Results:**

Compounds AC-R008, AC-M109, and AC-M110 inhibited TcME and *T. cruzi* intracellular growth. AC-R008 and AC-M110 exhibited good solubility at basic pH and favorable LogD_7.4_ values. AC-M110 exhibited high permeability with pH-dependent behavior in the PAMPA assay and intermediate values in the Caco-2 assay, while AC-R008 showed high permeability in both assays. However, both compounds presented short half-lives and high clearance in liver microsomal assays, consistent with extensive phase I metabolism. *In vivo*, oral administration resulted in plasma concentrations up to 1 µM after 1 h. In the Chagas disease animal model, AC-R008 and AC-M110 reduced parasitemia by 50% but did not reduce tissue parasite load.

**Conclusion:**

These findings demonstrate the potential of this sulfonamide scaffold while also underscoring metabolic instability and limited systemic exposure as major challenges. Future optimization efforts will focus on structural modifications and formulation strategies to enhance pharmacokinetics and therapeutic efficacy.

## Graphical Abstract

**Figure:**
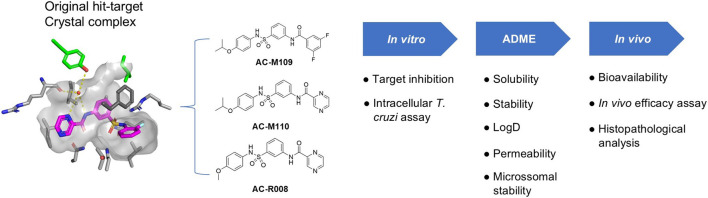

## 1 Introduction

Even more than a century since its discovery, Chagas disease (CD) remains a public health concern, resulting in approximately 12,000 deaths annually. It is estimated that around 7 million people worldwide are infected with *Trypanosoma cruzi* (World Health Organization, 2024). The disease progresses through clinically defined stages: an acute phase, typically characterized by mild symptoms and high parasitemia, and a chronic phase, which is predominantly asymptomatic. About 30% of chronically infected individuals eventually develop clinical manifestations such as cardiomyopathy or digestive mega syndromes (Chatelain and Scandale, 2020; Bivona et al., 2020).

Benznidazole (BNZ) and Nifurtimox (NFT) are currently the only approved drugs for treating CD. However, both are associated with adverse effects and cases of parasite resistance, and they often fail to fully control the infection, especially in the chronic phase, making the development of new, more effective drugs essential (Rial et al., 2023; Murta et al., 2024).

Advances in the understanding of parasite biology and its interaction with mammalian hosts have facilitated the identification of new molecular targets and chemical entities for drug discovery. In this context, the integration of high-throughput screening (HTS) and next-generation sequencing (NGS) technologies has greatly contributed to the chemical validation of promising targets such as cytochrome b (Khare et al., 2015) and proteasome (Khare et al., 2016). Although these advances in target identification are promising, the development of new drugs for CD remains complex, and few hit molecules have entered the pre-clinical research stage (Soeiro et al., 2024). It is currently widely accepted among researchers in the field that, for a molecule to progress to preclinical development, it must demonstrate an activity of ≤1 µM against the intracellular form of the parasite without inducing cytotoxicity in the host cell, possess favorable physicochemical properties, and, ideally, provide evidence of a mechanism of action associated with a *T. cruzi* target (Soeiro et al., 2024; de Oliveira et al., 2025).

In previous studies, our group identified a series of sulfonamides as inhibitors of the Malic Enzyme (TcME), an important enzyme in *T. cruzi* metabolism involved in redox balance and energy production (Ranzani et al., 2017; Cordeiro, 2019; Mercaldi et al., 2021). In the present study, a set of analogs was synthesized with a focus on improving the potency and drug likeness of original hits. These new compounds were then screened for enzymatic inhibition and efficacy against *T. cruzi* intracellular stage. With the target and activity established, the compounds were subsequently assessed for their *in vitro* ADME properties and *in vivo* bioavailability. Based on these findings, two sulfonamides were selected for further evaluation in a CD acute animal model.

## 2 Materials and methods

### 2.1 Organic synthesis of sulfonamides

Reagents and anhydrous solvents were purchased from Sigma-Aldrich (Brazil) and Labsynth (Brazil) (analytical grade) and were used without further purification unless specified otherwise. Reactions were monitored by thin-layer chromatography (TLC) on silica gel 60 F254 aluminum sheets and exposed to UV radiation, followed by treatment with adequate stains and heating. Chromatographic separations were carried out on Merck 60 silica gel (230–400 mesh). Melting points (m.p.) were recorded on a PF 1500 FARMA apparatus with a heating rate of 5 °C min-1 and were uncorrected. 1H NMR and 13C NMR data were recorded on a Varian 500 MHz spectrometer using TMS as the internal standard, or the residual nondeuterated solvent. Chemical shifts (δ) were expressed in ppm, and multiplicities were reported as singlet (s), doublet (d), double doublet (dd), triplet (t), double triplet (dt), quartet (q), heptet (h), multiplet (m), and triple triplet (tt). Coupling constants (J) are given in Hz and are uncorrected. High-resolution electrospray ionization mass spectrometry (HRMS-ESI) was performed on a BRUKER Impact II mass spectrometer.

#### 2.1.1 Method A

General Procedure for the Synthesis of nitro-*N*-phenylbenzenesulfonamides (5-8): Nitrobenzenesulfonyl chloride (1.0 equiv) and aniline (1.2 equiv.) were dissolved in anhydrous dichloromethane (451 mM) and cooled in an ice bath. Anhydrous pyridine (2.0 equiv) was added dropwise, and the reaction mixture was stirred at room temperature for 3 h. The progress of the reaction was monitored using TLC. After completion, the reaction mixture was diluted with CH_2_Cl_2_, washed sequentially with 1 M HCl solution and brine, then dried over anhydrous Na_2_SO_4_. The drying agent was filtered off, and the solvent was removed under reduced pressure. The crude product was subsequently applied to a chromatographic silica gel column, which was eluted using hexane/EtOAc (1:1, v/v). Fractions containing the product were identified via TLC, combined, and evaporated under reduced pressure to yield the desired product.

*N*-(4-methoxyphenyl)-3-nitrobenzenesulfonamide (5): The compound was synthesized using 3-nitrobenzenesulfonyl chloride (3.0 g, 13.53 mmol) and 4-methoxyaniline (1.99 g, 16.23 mmol) following Method A. The corresponding **5** (3.50 g, 83%), was obtained as an off white solid; mp: 134 °C–135 °C. ^1^H NMR (500 MHz, CDCl_3_): δ 8.58 (t, *J* = 2.0 Hz, 1H), 8.39 (ddd, *J* = 8.2, 2.3, 1.1 Hz, 1H), 7.97 (ddd, *J* = 7.8, 1.8, 1.1 Hz, 1H), 7.65 (t, *J* = 8.0 Hz, 1H), 7.03–6.96 (m, 2H), 6.82–6.76 (m, 2H), 6.57 (s, 1H), 3.77 (s, 3H). ^13^C NMR (126 MHz, CDCl_3_): δ 158.81, 148.35, 141.25, 133.01, 130.43, 127.64, 127.48, 126.21, 122.67, 114.92, 55.61. HRMS (ESI): *m*/*z* [M + H]^+^ calculated for C_13_H_13_N_2_O_5_S^+^: 309.0540, found: 309.0534.

*N*-(4-isopropoxyphenyl)-3-nitrobenzenesulfonamide (6): The compound was synthesized using 3-nitrobenzenesulfonyl chloride (3.44 g, 15.54 mmol) and 4-isopropoxyaniline hydrochloride (3.5 g, 18.65 mmol) following Method A. The corresponding 6 (4.182 g, 80%), was obtained as a light brown solid; mp:122 °C–123 °C. ^1^H NMR (500 MHz, DMSO-_d6_): δ 10.16 (s, 1H), 8.47–8.39 (m, 2H), 8.04 (ddd, *J* = 7.8, 1.8, 1.0 Hz, 1H), 7.84 (t, *J* = 8.0 Hz, 1H), 7.01–6.91 (m, 2H), 6.85–6.76 (m, 2H), 4.48 (p, *J* = 6.0 Hz, 1H), 1.18 (d, *J* = 6.0 Hz, 6H). ^13^C NMR (126 MHz, DMSO-_d6_): δ 155.28, 147.77, 140.94, 132.65, 131.24, 128.91, 127.38, 124.32, 121.43, 116.19, 114.48, 69.36, 21.70. HRMS (ESI): m/z [M + H]^+^ calculated for (C_15_H_20_N_3_O_5_S^+^): 354.1116, found: 354,1118.

*N*-(4-methoxyphenyl)-4-nitrobenzenesulfonamide (7): The compound was synthesized using 4-nitrobenzenesulfonyl chloride (3.0 g, 13.53 mmol) and 4-methoxyaniline (1.99 g, 16.23 mmol) following Method A. The corresponding **7** (3.81 g, 91%), was obtained as a gray solid; mp: 180 °C–182 °C. ^1^H NMR (500 MHz, DMSO-_d6_): δ 10.23 (s, 1H), 8.39–8.33 (m, 2H), 7.94–7.88 (m, 2H), 7.01–6.95 (m, 2H), 6.85–6.78 (m, 2H), 3.67 (s, 3H). ^13^C NMR (126 MHz, DMSO-_d6_): δ 156.98, 149.71, 144.89, 129.14, 128.29, 124.51, 124.09, 114.46, 55.16. HRMS (ESI): *m*/*z* [M + H]^+^ calculated for (C_13_H_13_N_2_O_5_S^+^): 309.0540, found: 308.9742.

*N*-(4-isopropoxyphenyl)-4-nitrobenzenesulfonamide (8): The compound was synthesized using 4-nitrobenzenesulfonyl chloride (1.33 g, 5.989 mmol) and 4-isopropoxyaniline hydrochloride (1.35 g, 7.19 mmol) following Method A The corresponding **8** (1.81 g, 90%), was obtained as a light pink solid; mp:150 °C–151 °C. ^1^H NMR (500 MHz, DMSO-_d6_): δ 10.21 (s, 1H), 8.36 (d, *J* = 8.8 Hz, 2H), 7.91 (d, *J* = 8.8 Hz, 2H), 6.95 (d, *J* = 9.0 Hz, 2H), 6.79 (d, *J* = 9.0 Hz, 2H), 4.54–4.16 (m, 1H), 1.19 (d, *J* = 6.0 Hz, 6H). ^13^C NMR (126 MHz, DMSO-_d6_): δ 155.17, 149.69, 144.95, 128.89, 128.27, 124.51, 124.05, 116.10, 69.32, 21.74. HRMS (ESI): *m*/*z* [M + NH_4_]^+^ calculated for (C_15_H_20_N_3_O_5_S^+^): 354.1118, found 354.1117.

#### 2.1.2 Method B

General Procedure for the Synthesis of amino-*N*-phenylbenzenesulfonamide (9-12): To nitrobenzenesulfonylamide (1.0 equiv) in AcOEt (135 mM) is added SnCl_2_.2H_2_O (5.0 equiv). The resulting mixture is heated at 80 °C for 4 h. The progress of the reaction was monitored using TLC. The reaction was cooled to room temperature, diluted with EtOAc, and the pH was adjusted to 7–8 by the addition of a 2M NaOH solution. The product was extracted into ethyl acetate, washed with water and brine, dried over anhydrous Na_2_SO_4_, and filtered, and the solvent was removed *in vacuo* to deliver the desired amine, which was used without further purification.

3-amino-*N*-(4-methoxyphenyl)benzenesulfonamide (9): The compound was synthesized using compound **5** (3.81 g, 12,336 mmol) following Method B. The corresponding **9** (2.90 g, 84%), was obtained as a gray solid; mp: 98 °C–99 °C. ^1^H NMR (500 MHz, DMSO-_d6_): δ 9.73 (s, 1H), 7.11 (t, *J* = 7.9 Hz, 1H), 7.01–6.94 (m, 2H), 6.89 (t, *J* = 2.3 Hz, 1H), 6.83–6.76 (m, 3H), 6.69 (ddd, *J* = 8.1, 2.3, 1.0 Hz, 1H), 5.52 (s, 2H), 3.66 (s, 3H). ^13^C NMR (126 MHz, DMSO-_d6_): δ 156.27, 149.19, 140.15, 130.50, 129.40, 123.06, 117.35, 114.18, 113.35, 111.27, 55.12. HRMS (ESI): *m*/*z* [M + NH_4_]^+^ calculated for (C_13_H_15_N_2_O_3_S^+^): 279.0798, found 279.0791.

3-amino-N-(4-isopropoxyphenyl)benzenesulfonamide (10): The compound was synthesized using compound 6 (3.93 g, 11.680 mmol) following Method A. The corresponding 10 (2.37 g, 66%), was obtained as a light pink solid; mp: 117 °C–119 °C. ^1^H NMR (500 MHz, DMSO-_d6_): δ 9.73 (d, *J* = 5.0 Hz, 1H), 7.12 (t, *J* = 7.9 Hz, 1H), 7.01–6.92 (m, 2H), 6.90 (t, *J* = 2.0 Hz, 1H), 6.82–6.73 (m, 4H), 6.72–6.66 (m, 2H), 5.54 (s, 0H), 4.47 (hept, *J* = 6.0 Hz, 1H), 1.19 (d, *J* = 6.0 Hz, 5H). ^13^C NMR (126 MHz, DMSO-_d6_): δ 154.41, 149.14, 140.23, 130.28, 129.38, 122.99, 117.36, 115.92, 113.36, 111.27, 69.29, 21.81. HRMS (ESI): m/z [M + H]^+^ calculated for (C_15_H_19_N_2_O_3_S^+^): 307.1111, found 307.1104.

4-amino-*N*-(4-methoxyphenyl)benzenesulfonamide (11): The compound was synthesized using compound **7** (3.00 g, 9.73 mmol) following Method A. The corresponding **11** (2.00 g, 73%), was obtained as off white solid; mp192 °C−194 °C. ^1^H NMR (500 MHz, DMSO-_d6_): δ 9.44 (s, 1H), 7.30 (dd, *J* = 8.8, 1.4 Hz, 2H), 6.95 (dd, *J* = 9.0, 1.1 Hz, 2H), 6.81–6.74 (m, 2H), 6.51 (dd, *J* = 8.8, 1.1 Hz, 2H), 5.91 (s, 2H), 3.66 (s, 3H). ^13^C NMR (126 MHz, DMSO-_d6_): δ 156.04, 152.61, 131.02, 128.63, 124.49, 122.86, 114.08, 112.49, 55.09. HRMS (ESI): *m*/*z* [M + H]^+^ calculated for (C_13_H_15_N_2_O_3_S^+^): 279.0798, found 279.0788.

4-amino-*N*-(4-isopropoxyphenyl)benzenesulfonamide (12): The compound was synthesized using compound **8** (1.70 g, 5.07 mmol) following Method A. The corresponding **12** (1.40 g, 89%), was obtained as a light pink solid; mp: 148 °C–149 °C. ^1^H NMR (500 MHz, DMSO-_d6_): δ 9.43 (s, 1H), 7.30 (d, *J* = 8.6 Hz, 2H), 6.92 (d, *J* = 8.8 Hz, 2H), 6.74 (d, *J* = 8.8 Hz, 2H), 6.51 (d, *J* = 8.6 Hz, 2H), 4.45 (h, *J* = 6.0 Hz, 1H), 1.19 (d, *J* = 6.0 Hz, 6H). ^13^C NMR (126 MHz, DMSO-_d6_): δ 154.19, 152.60, 130.82, 128.60, 124.56, 122.82, 115.88, 112.47, 69.27, 21.81 HRMS (ESI): *m*/*z* [M + H]^+^ calculated (C_15_H_19_N_2_O_3_S^+^): 307.1111 found 307.1106.

#### 2.1.3 Method C

General Procedure for the Synthesis of *N*-phenylsulfamoyl)phenyl)pyrazine-2-carboxamideamino Sulfonamides (13, 14, 15, and 16): In a screw-capped vial (20 mL), amino-*N*-phenylbenzenesulfonamide (5.29 mmol), pyrazine-2-carbonyl chloride (1.2 equiv), and ACN (352 mM) were combined at room temperature. Upon the addition of ACN to the medium, the material quickly dissolved, followed by the formation of a precipitate. TLC analysis confirmed the completion of the reaction. The vial’s contents were then mixed with water and extracted with EtOAc. The organic phase was dried over anhydrous Na_2_SO_4_, filtered, and adsorbed onto chromatographic silica via evaporation of the volatiles under reduced pressure. The crude product was subsequently applied to a chromatographic silica gel column, which was eluted using hexane/EtOAc (1:1, v/v), followed by a gradient to pure EtOAc. Fractions containing the product were identified via TLC, combined, and evaporated under reduced pressure to yield the desired product.

*N*-(3-(*N*-(4-methoxyphenyl)sulfamoyl)phenyl)pyrazine-2-carboxamide (13): The compound was synthesized using compound **9** (1.06 g 3.809 mmol), and pyrazine-2-carbonyl chloride (0.985g, 4.571 mmol) following Method C. The corresponding **13** (1.100 g, 75%), was obtained as white solid; mp: 176 °C–177 °C. ^1^H NMR (500 MHz, DMSO-_d6_): δ 11.06 (s, 1H), 10.00 (s, 1H), 9.30 (d, *J* = 1.5 Hz, 1H), 8.94 (d, *J* = 2.5 Hz, 1H), 8.82 (dt, *J* = 2.5, 1.3 Hz, 1H), 8.51 (d, *J* = 1.8 Hz, 1H), 8.00 (d, *J* = 8.2 Hz, 1H), 7.52 (t, *J* = 8.0 Hz, 1H), 7.43 (d, *J* = 7.8 Hz, 1H), 7.00 (dd, *J* = 9.0, 1.3 Hz, 2H), 6.80 (d, *J* = 9.0 Hz, 2H), 3.65 (s, 3H). ^13^C NMR (126 MHz, DMSO-_d6_): δ 162.19, 156.47, 147.88, 144.79, 144.18, 143.27, 140.10, 138.78, 130.04, 129.45, 124.37, 123.39, 122.24, 118.55, 114.28, 55.11. HRMS (ESI): *m*/*z* [M + NH_4_]^+^ calculated (C_18_H_20_N_5_O_4_S^+^): 402.1231 found 402.1236.

*N*-(3-(*N*-(4-isopropoxyphenyl)sulfamoyl)phenyl)pyrazine-2-carboxamide (14): The compound was synthesized using compound **10** (1.623 g, 5.29 mmol) and pyrazine-2-carbonyl chloride (1.367g, 6.35 mmol) following Method C. The corresponding **14** (1.270 g, 60%), was obtained as white solid; mp: 179 °C–180 °C. ^1^H NMR (500 MHz, DMSO-_d6_): δ 11.05 (s, 1H), 9.97 (s, 1H), 9.30 (d, *J* = 1.2 Hz, 1H), 8.94 (d, *J* = 2.5 Hz, 1H), 8.82 (dd, *J* = 2.5, 1.5 Hz, 1H), 8.50 (t, *J* = 1.8 Hz, 1H), 8.03–7.97 (m, 1H), 7.53 (t, *J* = 8.0 Hz, 1H), 7.44 (dd, *J* = 7.8, 1.0 Hz, 1H), 7.03–6.92 (m, 2H), 6.77 (d, *J* = 8.8 Hz, 2H), 4.46 (hept, *J* = 6.0 Hz, 1H), 1.17 (d, *J* = 6.0 Hz, 6H). ^13^C NMR (126 MHz, DMSO-_d6_): δ 162.16, 154.66, 147.87, 144.77, 144.16, 143.26, 140.13, 138.76, 129.82, 129.44, 124.36, 123.43, 122.20, 118.54, 116.03, 69.29, 21.75. HRMS (ESI): *m*/*z* [M + NH_4_]^+^ calculated (C_20_H_24_N_5_O_4_S^+^): 430.1544 found 430.1545.

*N*-(4-(*N*-(4-methoxyphenyl)sulfamoyl)phenyl)pyrazine-2-carboxamide (15): The compound was synthesized using compound **11** (0.14 g, 0.5 mmol) and pyrazine-2-carbonyl chloride (0.085 g, 0.6 mmol) following Method C. The corresponding **15** (0.100 g, 52%), was obtained as white solid; mp: 203 °C–204 °C. ^1^H NMR (500 MHz, DMSO-_d6_): δ 11.05 (s, 1H), 9.84 (s, 1H), 9.29 (d, *J* = 1.4 Hz, 1H), 8.95 (d, *J* = 2.4 Hz, 1H), 8.86–8.75 (m, 1H), 8.04 (d, *J* = 8.9 Hz, 2H), 7.67 (d, *J* = 8.9 Hz, 2H), 6.98 (d, *J* = 9.0 Hz, 2H), 6.80 (d, *J* = 9.0 Hz, 2H), 3.66 (s, 3H). ^13^C NMR (126 MHz, DMSO-_d6_): δ 162.30, 156.48, 147.97, 144.68, 144.23, 143.29, 141.88, 134.36, 130.19, 127.71, 123.39, 120.25, 114.29, 55.13. HRMS (ESI): *m*/*z* [M + H]^+^ calculated (C_18_H_17_N_4_O_4_S^+^): 385.0965 found 385.0970.

*N*-(4-(*N*-(4-isopropoxyphenyl)sulfamoyl)phenyl)pyrazine-2-carboxamide (16): The compound was synthesized using compound **12** (0.100 g, 0.32 mmol) and pyrazine-2-carbonyl chloride (0.089 g, 0.41 mmol) following Method C. The corresponding **16** (0.090 g, 69%), was obtained as white solid; mp 235 °C–237 °C. ^1^H NMR (500 MHz, DMSO-_d6_): δ 10.22 (s, 1H), 9.00 (s, 1H), 8.60–8.39 (m, 1H), 8.11 (d, *J* = 2.4 Hz, 1H), 8.02–7.89 (m, 1H), 7.21 (d, *J* = 8.7 Hz, 2H), 6.84 (d, *J* = 8.7 Hz, 2H), 6.12 (d, *J* = 8.9 Hz, 2H), 5.94 (d, *J* = 8.9 Hz, 2H), 3.63 (h, *J* = 6.0 Hz, 1H), 0.35 (d, *J* = 6.0 Hz, 6H). ^13^C NMR (126 MHz, DMSO-_d6_): δ 162.29, 154.65, 147.97, 144.68, 144.22, 143.28, 141.88, 134.42, 129.96, 127.69, 123.37, 120.23, 116.00, 69.29, 21.78. HRMS (ESI): *m*/*z* [M + H]^+^ calculated (C_20_H_21_N_4_O_4_S^+^): 413.1278 found 413.1261.

#### 2.1.4 Method D

General Procedure for the Synthesis of 3,5-difluoro-(*N*-phenylsulfamoyl)phenyl)benzamide (17 and 18): Amino-*N*-phenylbenzenesulfonamide (2.61 mmol), 3,5-difluorobenzoyl chloride (1.2 equiv), ACN (326 mM), and *N*-methylmorpholine (2 equiv) were mixed at room temperature for 30 min. The progress of the reaction was monitored using TLC. Upon completion of the reaction, the mixture was quenched with water. The aqueous phase was extracted with EtOAc. The organic extracts were combined, dried over anhydrous Na_2_SO_4_, filtered, and absorbed onto chromatographic silica via evaporation of the volatiles under reduced pressure. The crude product was subsequently applied to a chromatographic silica gel column, which was eluted using hexane/EtOAc (1:1, v/v), followed by a gradient to pure EtOAc. Fractions containing the product were identified via TLC, combined, and evaporated under reduced pressure to yield the desired product.

3,5-difluoro-*N*-(3-(*N*-(4-isopropoxyphenyl)sulfamoyl)phenyl)benzamide (17): the compound was synthesized using compound **10** (0.800 g, 2.61 mmol) and 3,5-difluorobenzoyl chloride (0.576g, 3.25 mmol) following Method D. The corresponding **17** (0.812 g, 70%), was obtained as white solid; mp: 199 °C–201 °C. ^1^H NMR (500 MHz, DMSO-_d6_): δ 10.59 (s, 1H), 9.96 (s, 1H), 7.94 (d, *J* = 8.6 Hz, 0H), 7.72–7.64 (m, 2H), 7.58–7.50 (m, 2H), 7.44 (d, *J* = 7.9 Hz, 1H), 7.00–6.91 (m, 3H), 6.81–6.73 (m, 2H), 4.47 (hept, *J* = 6.0 Hz, 1H), 1.18 (d, *J* = 6.0 Hz, 6H). ^13^C NMR (126 MHz, DMSO-_d6_): δ 163.17 (d, *J*_*C-F*_ = 12.6 Hz), 161.20 (d, *J*_*C-F*_ = 12.9 Hz), 154.73, 140.14, 139.16, 137.80, 129.77, 129.57, 123.95, 123.54, 122.03, 118.33, 116.05, 111.31 (d, *J*_*C-F*_ = 6.3 Hz), 111.14 (d, *J*_*C-F*_ = 6.4 Hz), 107.54, 107.33, 69.31, 21.7. HRMS (m/z + H^+^): obs.: 447.1165; calc.: 447.1185 (C_22_H_21_F_2_N_2_O_4_S^+^).

3,5-difluoro-N-(4-(N-(4-methoxyphenyl)sulfamoyl)phenyl)benzamide (18): The compound was synthesized using compound **11** (0.140 g, 0.5 mmol) and 3,5-difluorobenzoyl chloride (0.105 g, 0.6 mmol) following Method D. The corresponding **18** (0.146 g, 75%), was obtained as white solid; mp: 250 °C–252 °C. ^1^H NMR (500 MHz, DMSO-_d6_): δ 10.64 (s, 1H), 9.83 (s, 1H), 7.95–7.84 (m, 2H), 7.73–7.63 (m, 4H), 7.55 (tt, *J* = 9.1, 2.3 Hz, 1H), 7.00–6.96 (m, 2H), 6.82–6.78 (m, 2H), 3.66 (s, 3H). ^13^C NMR (126 MHz, DMSO-_d6_): δ 163.409 (as the two peaks of C-F carbons overlapped so it was not possible to see the triplet for this carbon), 162.16 (dd, *J*_C-F_ = 247.5, 12.6), 161.12, 156.47, 142.30, 137.85, 134.16, 130.17, 127.79, 123.43, 119.98, 111.38 (dd, *J*_C-F_ = 7.5, 6.6), 107.38 (t, *J*_C-F_ = 26.0 Hz), 55.12. HRMS (ESI): *m*/*z* [M + H]^+^ calculated (C_20_H_17_F_2_N_2_O_4_S^+^): 419.0872 found 419.0874.

### 2.2 Malic enzyme inhibition assay

The TcME inhibition assay was performed as previously described, with minor modifications (Mercaldi et al., 2021). Briefly, a coupled assay with the enzyme diaphorase and resorufin fluorescence (Ex 545 nm/Em 600 nm) was recorded every 30 s for 6 min in a CLARIOstar plate reader (BMG LabTech). The final concentrations of reagents in the assay were: 0.02 mM NADP+, 0.4 mM L-aspartate, 0.45 nM TcMEc, 1 U/mL diaphorase, and 10 μM resazurin, all in 50 mM Tris-HCl buffer containing 50 mM NaCl and 2 mM MnCl_2_ at pH 7.5. The reaction was initiated by adding 1.32 mM L-malate.

### 2.3 *T. cruzi* intracellular image-based assay

Tested compounds were dissolved and serially diluted in dimethylsulfoxide (DMSO) using a half-log dilution across six concentrations. Intermediate plates were prepared by diluting compounds 1:25 in DMEM (Dulbecco’s Modified Eagle Medium) with 10% FBS (Fetal Bovine Serum). The assay was based on Fredo Naciuk et al. (2020), with minor modifications. Briefly, *T. cruzi* trypomastigotes (from LLC-MK2 cultures, 6 days post-infection) were used to infect H9c2 rat cardiomyoblasts overnight (16–18 h) at MOI 1. After washing with PBS, cells were trypsinized and resuspended in DMEM at 2.3 × 10^4^ cells/mL. Then, 65 μL per well was plated in 384-well plates and incubated for 24 h (37 °C, 5% CO_2_, >95% humidity). Subsequently, 7.5 μL of compound solution was added (final [DMSO] 0.4%). After 72 h of treatment, cells were fixed with 4% PFA and stained with Hoechst 33342 (4 μg/mL). Six images per well were acquired using the Operetta system (PerkinElmer) and analyzed with Columbus software to quantify infected cells (≥3 cytoplasmic parasite spots).

### 2.4 Kinetic solubility and chemical stability

The compounds AC-M110 and AC-R008 were prepared at a concentration of 5 µM in 0.4% DMSO and evaluated at pH 1.7 (Clark-Lubs buffer, 0.2 M), pH 7.4 (sodium phosphate buffer, 0.1 M), and pH 8.9 (Tris buffer adjusted with 2 M hydrochloric acid). These conditions simulate stomach acidity, plasma neutrality, and intestinal basicity.

For the solubility assay, the solutions were maintained at 25 °C (Thermal Mixer, Eppendorf), and samples (200 μL) were collected at 0 and 1.5 h. For the chemical stability experiment, the compounds were evaluated under the same conditions with an additional incubation at 37 °C for a period of 24 h. The analysis was performed using an analytical method developed and validated by high-performance liquid chromatography with UV detection (HPLC-UV). Compounds were quantified in different pH media by comparing the areas where the substances were completely soluble with those in the reaction media. The experiments were performed in a triplicate.

### 2.5 Lipophilicity assay (LogD at pH 7.4)

For this assay, an aqueous phosphate buffer solution at pH 7.4, saturated with n-octanol, was prepared, along with an n-octanol solution saturated with the aqueous phase at pH 7.4. Both solutions were mechanically stirred (using a magnetic stir bar and a Corning magnetic stirrer) at room temperature for 24 h, followed by a 24-hour resting period to ensure complete phase separation. Different phase ratios were prepared from these solutions in the following aqueous phase/n-octanol ratios: 1:1, 1:10, 1:100, and 1:300, to ensure that the substances were evaluated under non-saturating conditions. Stock solutions of each compound were prepared in DMSO at a concentration of 20 mM. From these stock solutions, standard solutions at a concentration of 30 µM in phosphate buffer pH 7.4 were obtained and used in each partition system, performed in triplicate. To reach equilibrium, the vials containing the samples were shaken for 90 min at room temperature using a roller shaker (Basic First Lab Orbital). After this period, the phases were separated, and the aqueous phase was analyzed using an HPLC-UV system to quantify the compounds, according to the chromatographic conditions and parameters previously adjusted (EPA, 1996).

### 2.6 Caco-2 permeability assay

Caco-2 cells (2.5 × 10^5^) were cultured on 12 mm diameter filters with DMEM-PEST medium in an incubator at 37 °C and 10% CO_2_. Monolayer formation was monitored through transepithelial electrical resistance (TEER). After monolayer formation (approximately 21 days), the compounds AC-M110, AC-R008, and the reference compound (Verapamil) were added to the apical region at a concentration of 0.1 mM, diluted in HBSS (Hanks’ Balanced Salt Solution) medium. The assay was conducted under orbital shaking at 37 °C. Samples from the basolateral region were collected at 0, 10, 30, 60, 90, and 120 min. In the apical region, samples were collected at 0 and 120 min. Compound quantification was performed using the HPLC-UV method, following the previously adjusted chromatographic conditions and parameters. At the end of the experiment, monolayer integrity was confirmed by measuring TEER. The apparent permeability coefficient (Papp) was calculated using the following formula:

$$Papp=\left(\frac{dQ}{dt}\right)x\left(\frac{1}{AC0}\right)$$

where dQ/dt is the steady-state flux (dQ is the total final concentration on the basolateral side in μmol, and dt is the total time in seconds), A is the surface area of the filter (1.2 cm^2^), and C0 is the concentration in the donor chamber (100 μM).

### 2.7 PAMPA assay

The parallel artificial membrane permeability assay (PAMPA) was performed using a 96-well pre-coated plate system (Corning® BioCoat™ PAMPA Plate System). Verapamil (reference compound) and test compounds AC-M110 and AC-R008 were prepared from 10 mM DMSO stock solutions diluted in phosphate-buffered saline (PBS, pH 5.5 and pH 7.4) to a final concentration of 100 μM, ensuring a DMSO content <1%. A total of 200 µL of each solution was added to donor wells, while 300 µL of PBS was placed in the corresponding acceptor wells. The plates were assembled and incubated at 37 °C for 5 h. Aliquots of the initial donor solution (T0) were collected before incubation and stored at −20 °C. At the end of the incubation, samples from donor and acceptor compartments were transferred to tubes containing 30% (v/v) acetonitrile and processed under identical conditions. Compound concentrations in donor, acceptor, and T0 samples were quantified by high-performance liquid chromatography with ultraviolet detection (HPLC-UV) using validated chromatographic conditions. The apparent permeability coefficient (Papp) was calculated using the standard PAMPA equation, shown below. All experiments were performed in a trplicate. Calibration curves (2.3–150 µM) were used to quantify compound concentrations in both donor and acceptor compartments (upper and lower fractions) at each pH. According to the literature (Kerns and Di, 2008; Ogilvie et al., 2008; Obach, 1999), compounds are classified as highly permeable when Papp exceeds 4.0 × 10^−6^ cm/s in the acceptor compartment.

The Papp equation is:

$$Papp=-\left(V_{D}+V_{A}\right)/At\;\left(V_{D}+V_{A}\right)\ln\;\left(1-C_{A}\;\left(t\right)/C_{eq}\right)$$

Where: Papp = apparent permeability coefficient (cm/s); V_D_ = donor volume (cm^3^); V_A_ = acceptor volume (cm^3^); A = effective membrane area (cm^2^); t = incubation time (s); C_A_ (t) = compound concentration in the acceptor at time t; C_eq_ = equilibrium concentration calculated from C_D_ (t) and C_A_ (t).

### 2.8 Metabolic stability

The *in vitro* metabolic stability of the compound was evaluated in pooled human liver microsomes (HLM, 20 mg/mL), Sprague-Dawley rat liver microsomes (RLM, 20 mg/mL), and CD-1 mouse liver microsomes (MLM, 20 mg/mL), all purchased from GIBCO (Thermo Fisher Scientific). NADPH regenerating system (GentestTM) was prepared in potassium phosphate buffer (100 mM, pH 7.4). The compounds AC-M110, AC-R008 and Verapamil were evaluated at a concentration of 1 μM. The assay was initiated by adding microsomes (0.25 mg/mL), and samples were collected at 0, 1,3, 5, 15, 30, 45 and 60 min of reaction. The reaction was stopped by adding acetonitrile, followed by vortex homogenization, sonication for 5 min, and centrifugation at 10,000 g for 5 min at 4 °C. The supernatants were transferred to glass vials for LC-MS/MS analysis. The half-life was estimated based on the area under the curve (AUC) through regression analysis of the concentration decay over time. This calculation was performed using the following formula:

$$t_{\frac{1}{2}}=\mathrm{LN}2/K_{el}$$

Where LN is the neperian logarithm, and K is the slope of the regression of the LN of AUC as a function of time. Then, the microsomal intrinsic clearance (CL_int, mic_) was calculated using the following equation:

$${CL}_{\mathrm{int}.mic}=\frac{\mathrm{LN}2}{t_{\frac{1}{2}}}\;x\;\frac{VI}{PM}$$

Where V is the incubation volume (µL), and PM is the microsomal protein concentration (mg). This intrinsic clearance can be extrapolated to the whole liver (CL_int,hep_) using the following equation:

$${CL}_{\mathrm{int}.hep}={CL}_{\mathrm{int}.mic}\;x\;ML\;x\;LB$$

ML is the microsomal yield, and LB is the liver weight (g) per kilogram of body weight. A microsomal yield of 45 mg per 1 g of liver, a blood flow of 90 mL/min/kg, and a liver weight of 90 g per 1 kg of body weight were considered for mice.

To ensure the linearity of the elimination phase and to avoid the influence of points outside the log-linear range, the points deviated from the expected first-order kinetic profile were excluded. The supernatants were analyzed by UPLC-Xevo-TQ-XS from Waters. Chromatographic separation was performed on an Acquity UPLC BEH C18 column (2.1 × 100 mm, 1.7 µm particle size), maintained at 40 °C. The mobile phases were (A) water with 0.1% formic acid and (B) acetonitrile with 0.1% formic acid. A binary gradient was applied as follows: 0–1 min, 65% A; 1–4 min, 5% A; 4.5–5 min, 65% A. The flow rate was 0.4 mL/min, and the injection volume was 1 μL. The total run time was 5 min. Compound AC-M110 was monitored in MRM mode using the precursor ion m/z 413.07, with product ions m/z 108.88, 150.95, and 371.01. AC-R008 was monitored using the precursor ion m/z 384.98 and product ions m/z 107.97 and 122.93. Verapamil was monitored using the precursor ion m/z 455.28 and product ions m/z 149.95, 164.97, and 303.11.

### 2.9 *In vivo* studies

For the *in vivo* assays, female BALB/c mice, aged between 8 and 9 weeks and weighing approximately 25 g, were used. The animals were supplied by CEMIB/Unicamp and acclimated to the CNPEM animal experimentation facility before the experiment. There, they were kept under standard conditions on a 12-hour light-dark cycle in a temperature-controlled environment (22 °C–24 °C), with food and water provided *ad libitum*. This study was approved by the institutional Animal Ethics Committee CEUA-CNPEM (protocol numbers 114 and 115) and conducted in accordance with the ARRIVE (Animal research: Reporting of *In Vivo* Experiments) guidelines as described at: https://arriveguidelines.org.

#### 2.9.1 Oral bioavailability

The compounds AC-M110 and AC-R008 were suspended in a vehicle solution of 5% ethanol, 5% Tween 80, 45% PEG 400, and 45% PBS (Gad et al., 2016). After preparation, three animals received a single oral dose of 50 mg/kg for each compound via gavage. The control group received only the vehicle. Blood samples were collected at 5-, 15-, 30-, 45-, and 60-minute post-administration using submandibular vein puncture, retro-orbital collection, and intracardiac (terminal) puncture. The mice were maintained under inhalation anesthesia with 3% isoflurane throughout the sampling period to ensure animal welfare. Blood samples were transferred to heparin-containing tubes and processed by centrifugation (4,000 rpm, 10 min, 4 °C) to separate the plasma. The obtained plasma was transferred to clean vials and stored at −80 °C until analysis. Compound quantification was performed using ultra-performance liquid chromatography coupled with tandem mass spectrometry (UPLC-MS/MS).

#### 2.9.2 *In vivo* efficacy assay

The *T. cruzi* strain Dm28c (DTU TcI) was maintained in monkey kidney epithelial cell cultures (LLC-MK2) grown in DMEM medium supplemented with 2% FBS, 4.5 g L^-1^ de glucose, and 1% penicillin/streptomycin (P/S). For the experimental infection, twenty animals were inoculated with 1 × 10^5^ bloodstream trypomastigotes via the intraperitoneal and were randomly assigned into groups of five animals each. Briefly, a pilot experiment was conducted only with control groups and monitored for 30 days. The selected compounds, AC-M110 and AC-R008, were administered by gavage in a suspension volume of 100 µL using a previously determined vehicle (5% ethanol, 5% Tween 80, 45% PEG400, and 45% PBS) at a dosage of 150 mg/kg *semel in die* (SID). For the positive control, BNZ was used at 50 mg/kg (SID). Treatment with sulfonamides and BNZ began on the 4th day post-infection (dpi) and lasted for 14 days. Animals in the negative control group received vehicle only, administered by the same route and dosing schedule as the treatment groups. Uninfected age-matched mice were maintained under identical conditions. All animals were euthanized on day 20, and biological materials were collected for analysis.

Parasitemia was assessed three times per week using fresh blood collected from the mice’s tail (5 µL), and the number of parasites was estimated using the Neubauer chamber following the Pizzi-Brenner method. Parasite burden in heart, spleen, and colon samples was quantified by real-time quantitative PCR. Tissue DNA was extracted from 20 mg samples using the commercial “Wizard® Genomic DNA Purification Kit, Promega” following the manufacturer’s recommendations. qPCR reactions were carried out using SYBR® Green PCR Master Mix (Applied Biosystems) according to manufacture recommendations, with 75 ng of template DNA and 500 nM of *T. cruzi*-specific primers 5′-GCT​CTT​GCC​CAC​AAG​GGT​GC-3′ (TCZ-F), 5′-CCA​AGC​AGC​GGA​TAG​TTC​AGG-3′ (TCZ-R). For parasite load quantification, standard curves were generated for each tissue by adding 1 × 10^6^ *T. cruzi* epimastigotes from cell culture into 20 mg of a non-infected mouse tissue and performing a 1:10 serial dilution in Milli-Q water.

For histopathological analysis, animal hearts were fixed in 10% formalin solution for 24h, dehydrated in increasing ethanol series (70%–99.8%), clarified in xylene, and embedded in histological-grade paraffin. Histological sections with 4-µm thick were cut in a rotary microtome (Leica Multicut, Wetzlar, Germany), stained with hematoxylin and eosin, and mounted on histological slides with Entelan (Merk, Darmstadt, Germany). The organs were observed, and digital images were captured by using a bright field photomicroscope (Axio Scope A1, Carl Zeiss, Oberkochen, Germany). Histopathological analysis was performed qualitatively based on the observation and characterization of classical alterations in each of the organs investigated.

### 2.10 Data analysis

Data analysis and interpretation were performed using GraphPad Prism 9.0 (GraphPad Software, Inc., La Jolla, CA, USA). Results are expressed as mean ± standard deviation (SD). EC_50_ and IC_50_ values were calculated by the nonlinear fit of normalized data to a sigmoidal curve with variable slope. Statistical analysis of solubility and stability assays was performed using two-way ANOVA followed by Tukey’s multiple comparison test. The animal assay data were analyzed using one-way ANOVA followed by Dunnett’s multiple comparison test. Statistical significance was set at p ≤ 0.05.

## 3 Results

### 3.1 Synthesis of new sulfonamides

**FIGURE 1:**
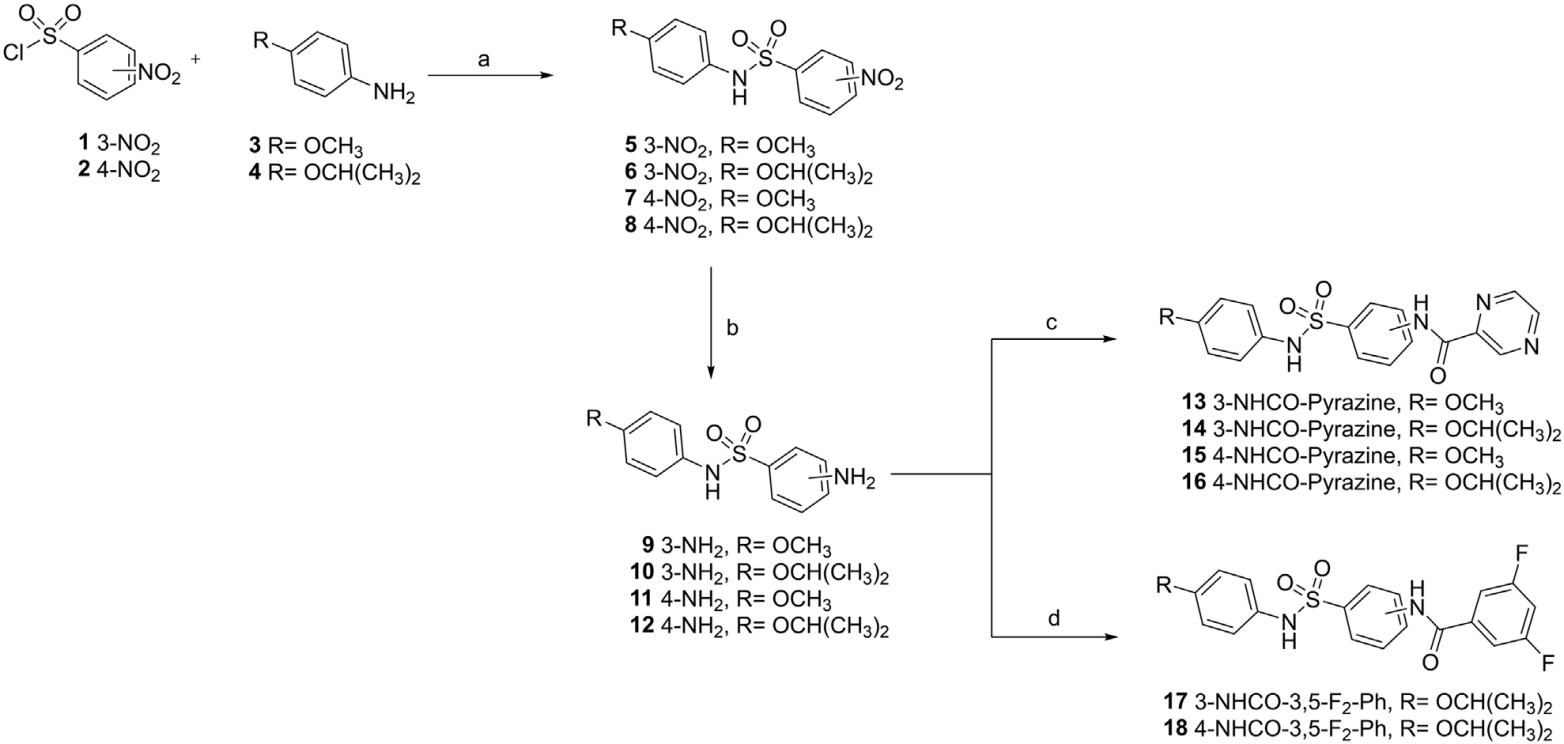
Synthetic route to prepare new sulfonamides. The route comprises four steps with different reagents and conditions: **(a)** anhydrous dichloromethane (DCM), pyridine, 0 °C−r.t., 3 h, 70%–91%; **(b)** ethyl acetate (AcOEt), SnCl2 2H2O, reflux, 4 h; 66%–89%; **(c)** pyrazine-2-carbonyl chloride, acetonitrile (ACN), r.t., the reaction occurred instantly, 36%–83%; **(d)** 3,5-difluorobenzoyl chloride, ACN (8 mL), and N-methylmorpholine, 0.5 h, 52%–75%.

The sulfonamides were synthesized following a previously described three-step procedure, with small modifications (Mercaldi et al., 2021). Briefly, the first step consists in the construction of diaryl sulfonamides from 3- or 4-nitrobenzenesulfonyl chlorides with the corresponding anilines (R = OMe and R = OiPr); then, the nitro group was reduced, leading to the formation of anilines; finally, the resulting aniline intermediates were coupled with pyrazine-2-carbonyl chloride or 3,5-difluorobenzoyl chloride. At this stage, the reactions between anilines and pyrazine-2-carbonyl chloride occurred instantly upon the addition of acetonitrile to the reaction medium (Figure 1). The acetonitrile dissolved the reagents, followed by rapid precipitation. The compounds were synthesized on a 1-gram scale for *in vivo* testing, and UPLC analysis revealed high sample purity.

### 3.2 *In vitro* activity profile of new sulfonamides

**FIGURE 2:**
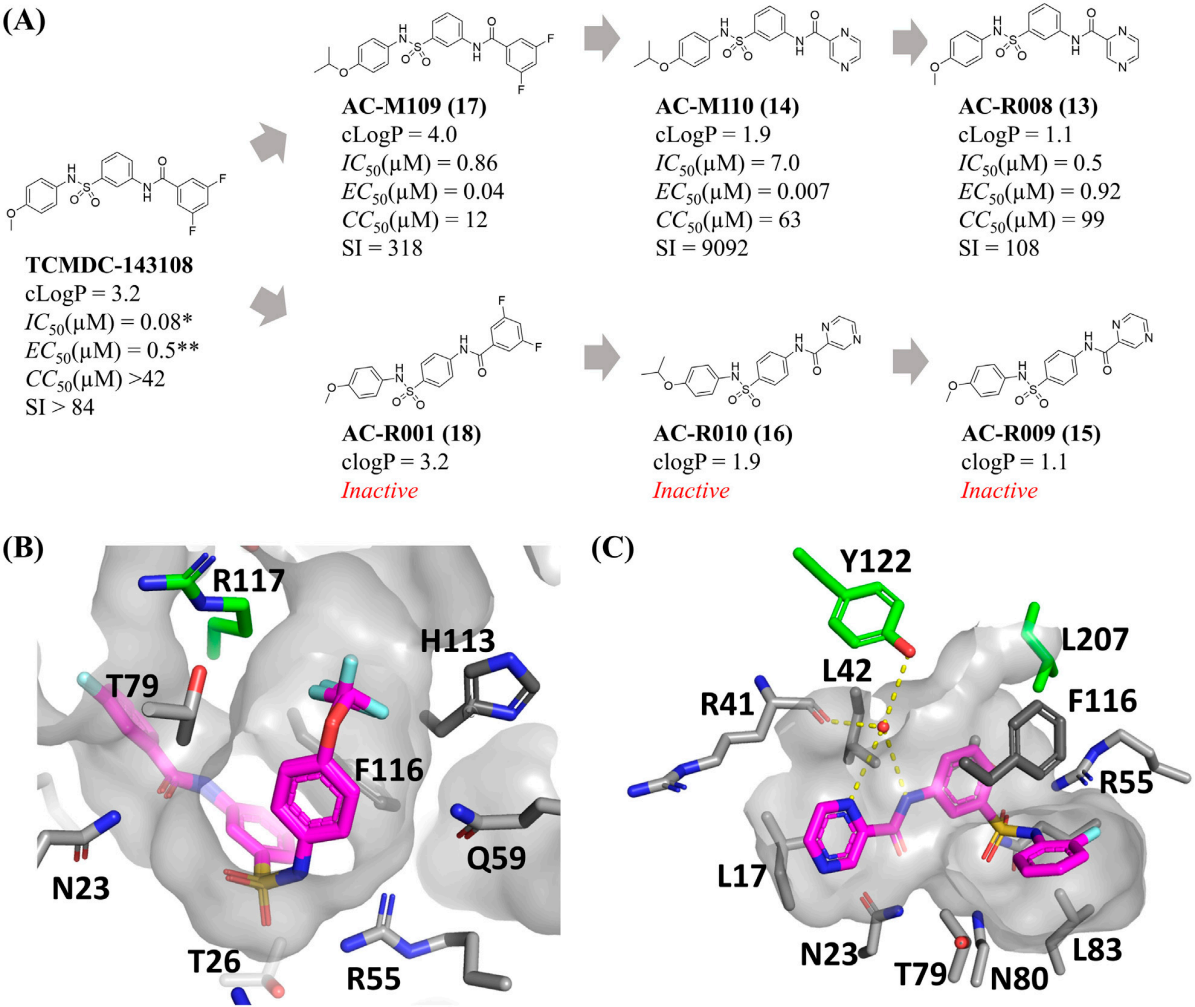
Activity profile of new sulfonamides and structure-based rational for ligand design. **(A)** Chemical structures, properties, and biological activity of the compounds studied. **(B,C)** Allosteric binding pocket of the sulfonamides in the *T. cruzi* malic enzyme, highlighting difluoro- and trifluoro-methoxy groups on the phenyl ring near the sulfonamide, as well as the aniline ring bound to a water molecule that also interacts with residues Y122 and R41. (cLogP) LogP calculated, (IC_50_) concentration necessary to inhibit 50% of *T. cruzi* Malic enzyme enzymatic activity, (EC_50_) host cells infected by *T. cruzi* amastigotes, (CC_50_) total host cells, (SI) Selectivity index. All the experiments’ activity was determined from dose-response assays conducted in three biological replicates. TCMDC-143108 activity parameters were reproduced from Mercaldi et al. (2021)* and Peña et al. (2015)**. Panel B was prepared using the PDB ID 6W56 and 6W59, while PDB ID 6W49 was used to prepare the figure on panel C.

Six new sulfonamides were synthesized and tested for inhibition of TcME and against the intracellular form of *T. cruzi*, infecting h9c2 rat cardiomyoblast (Figure 2A). The development of novel sulfonamide analogs was directed by previous crystallographic analyses of the TcME enzyme in complex with a variety of inhibitors (Mercaldi et al., 2021). These structural insights highlighted the potential for strategic modifications at specific positions on the phenyl ring. In particular, the data indicated that an isopropoxy substituent could serve as an effective replacement for the difluoro- and trifluoro-methoxy groups commonly employed in earlier analogs. This substitution was shown to maintain the critical complementarity between the ligand and the enzyme’s binding site, ensuring effective interaction and preserving biological activity (Figure 2B). Replacing the methoxy group with an isopropoxy group in the reference compound TCMDC-143108 yielded AC-M109, which demonstrated enhanced activity against intracellular forms of *T. cruzi,* along with an increased cLogP value (Figure 2A). Building on the foundation of previously identified inhibitors that utilized a scaffold featuring a pyrazine ring, further structural modifications were undertaken to enhance the pharmacological profile of the compounds. Specifically, the difluorobenzene group present in AC-M109 was substituted with a pyrazine ring, resulting in the generation of AC-M110 (Figure 2C). This targeted replacement aimed to adjust the physicochemical properties of the molecule while retaining its antiparasitic potency. The introduction of the pyrazine ring into the scaffold led to a notable reduction in the compound’s calculated logarithm of partition coefficient (cLogP), bringing it down to 1.9. This change signifies improved solubility and potentially greater suitability for oral administration. Importantly, AC-M110 maintained EC_50_ values within the nanomolar range, indicating that its efficacy against the intracellular forms of *T. cruzi* remained robust despite the structural alteration. However, the modification was associated with an increase in IC_50_ values (Figure 2A), suggesting a decrease in the compound’s inhibitory potency against the target enzyme. Further modification involved synthesizing AC-R008 by retaining the pyrazine ring and reintroducing the methoxy group. This compound exhibited low lipophilicity and preserved the capacity to inhibit TcME, but an increased EC_50_ value. Still aiming to explore new arrangements of this scaffold without altering the chemical composition and lipophilicity, we synthesized molecules AC-R009 and AC-R010, where the position of the pyrazine-carboxamide group was changed from *meta* to *para.* This modification was designed to explore cavities near the binding site or a stacking interaction with Tyr122 (Figure 2C), without altering the composition and physicochemical properties. Remarkably, these modifications abolished the biological activity of both compounds (Figure 2A). Similar results were obtained for compound AC-R001, an analogue of TCMDC-143108 with the difluorobenzene in the *para* orientation. Synthetic intermediates obtained before the introduction of the third aromatic group in *meta* orientation (compounds 5, 6, 9, and 10) were not active against TcME or *T. cruzi* intracellular forms.

### 3.3 Solubility and chemical stability at physiological pHs

**FIGURE 3:**
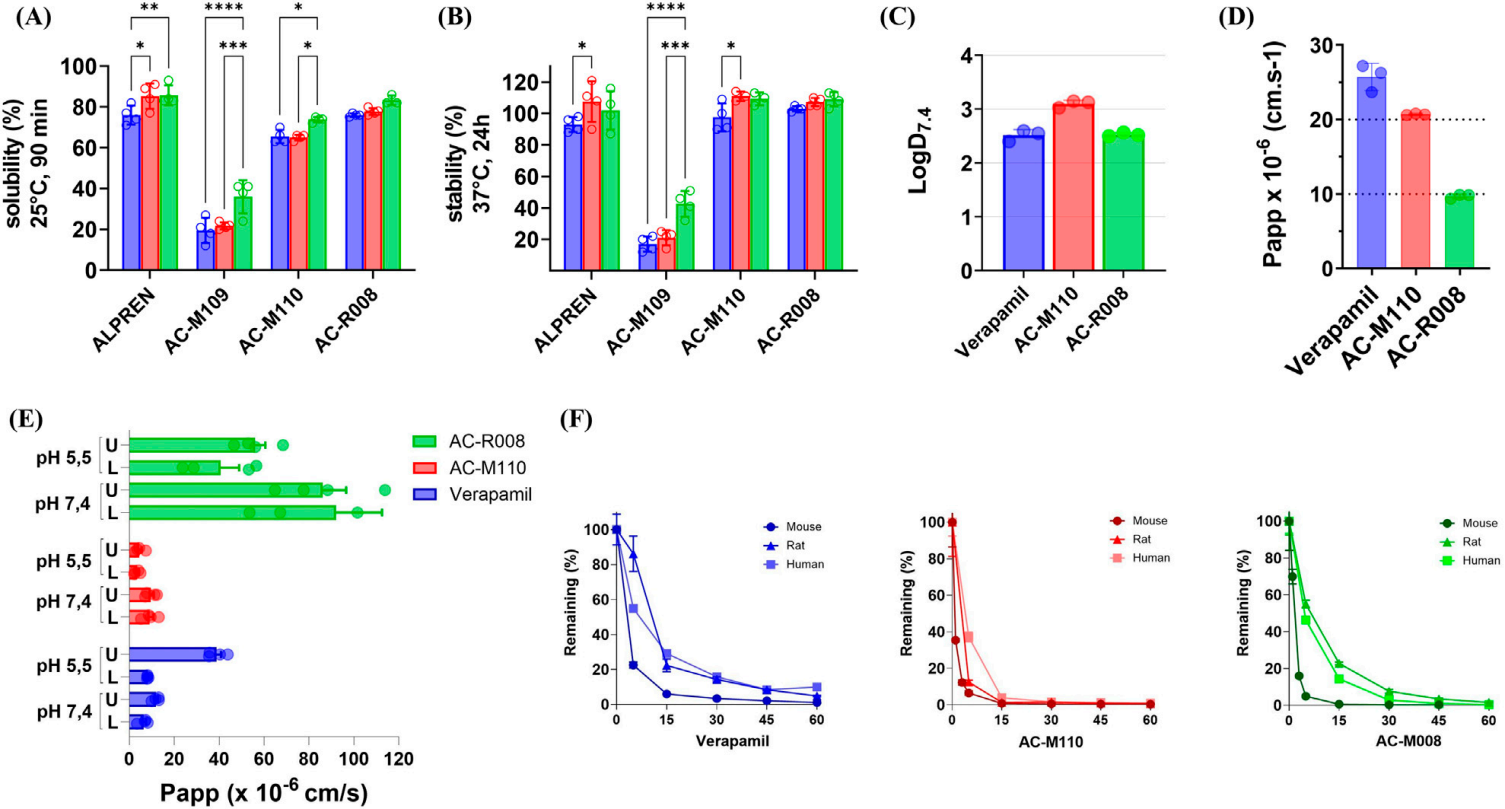
*In vitro* ADME. **(A)** Kinetic solubility and **(B)** chemical stability were evaluated at three pH values: 1.7 (blue bars), 7.4 (green bars), and 8.9 (blue bars). **(C)** LogD_7.4_, **(D)** Caco-2 permeability assay, and **(E)** PAMPA permeability assay, compounds were introduced into the donor compartment on either the upper (U) or lower (L) side. The x-axis shows permeability values (cm/s), and the y-axis lists the compounds tested. Measurements were taken after 6 h of incubation. **(F)** Microsomal stability profiles of Verapamil, AC-M110, and AC-R008 in liver microsomes from mouse, rat, and human. The percentage of parent compound remaining over time was determined by LC-MS/MS. Data represent the mean ± standard deviation (SD) of three independent experiments (n = 3). Statistical significance was assessed using two-way ANOVA followed by Tukey’s multiple comparison test. Adjusted p values are indicated by asterisks (*p ≤ 0.05; **p ≤ 0.01; ***p ≤ 0.001; ****p ≤ 0.0001).

Solubility was assessed at 25 °C at 0 and 90 min, while chemical stability was measured at 37 °C at 0, 1.5 (Supplementary Figure S1), and 24 h across different pH values simulating gastrointestinal and bloodstream conditions. In the solubility assays, AC-M110 retained 65% solubility under acidic and neutral pH, increasing to 74% under basic pH. AC-R008 exhibited superior behavior, achieving 75% solubility under all three pH conditions (Figure 3A). Both compounds displayed solubility profiles comparable to Alprenolol (reference drug). In contrast, AC-M109 demonstrated limited solubility, ranging from 19% to 36% across pH conditions. Regarding chemical stability, AC-M110 and AC-R008 remained stable above 80% across all pH conditions, comparable to Alprenolol, whereas AC-M109 showed poor stability, not exceeding 40% under basic pH (Figure 3B). Based on these results, AC-M110 and AC-R008 were prioritized for subsequent studies, while AC-M109 was excluded due to inadequate solubility and stability.

### 3.4 Structural modification improves compounds cell permeability but increases metabolic liability in liver microsomes

To gain an initial understanding of the cellular permeability of AC-M110 and AC-R008, we assessed lipophilicity (LogD_7.4_), stability in HBSS buffer (Supplementary Figure S2), and apparent permeability (Papp) using both Caco-2 and PAMPA assays. The LogD_7.4_ values of both compounds were within the optimal range (1–3), with AC-M110 at the upper limit, indicating higher lipophilicity than AC-R008 (Figure 3C; Table 1).

**TABLE 1:** ADME parameters of compounds AC-M110, AC-R008, and the positive control Verapamil.

Parameters	Verapamil	AC-M110	AC-R008
LogD	2.50	3.00	2.50
Caco-2 P_app_ (cm.s^−1^)	25.7 × 10^−6^	20.7 × 10^−6^	9.71 × 10^−6^
PAMPA pH 5,5 P_app_ (cm/s) - upper	38.8 × 10^−6^	4.4 × 10^−6^	56.0 × 10^−6^
PAMPA pH 5,5 P_app_ (cm/s) - lower	8.1 × 10^−6^	3.4 × 10^−6^	40.5 × 10^−6^
PAMPA pH 7,4 P_app_ (cm/s) - upper	11.9 × 10^−6^	9.6 × 10^−6^	86 × 10^−6^
PAMPA pH 7,4 P_app_ (cm/s) - lower	6.4 × 10^−6^	9.1 × 10^−6^	92 × 10^−6^
T_1/2_ (min) - mouse	19	2	3
CL int. mic. (µL·min^−1^·mg^−1^)	30	339	200
CL int. hep. (µL·min^−1^·kg^−1^)	120	1,372	811
T_1/2_ (min) rat	20	9	10
CL int. mic. (µL·min^−1^·mg^−1^)	27	61	55
CL int. hep. (µL·min^−1^·kg^−1^)	55	110	99
T_1/2_ (min) human	27	6	7
CL int. mic. (µL·min^−1^·mg^−1^)	20	97	78
CL int. hep. (µL·min^−1^·kg^−1^)	18	87	70

Permeability was evaluated under physiologically relevant conditions (pH 5.5 and 7.4), using verapamil as a reference (Figures 3D,E; Table 1). In the Caco-2 model, AC-R008 showed a Papp value near the lower limit for moderate permeability, whereas AC-M110 reached the upper limit, approaching high permeability classification. In the PAMPA assay, all compounds met the criteria for high permeability. Verapamil displayed values between 6.4–11.9 × 10^−6^ cm/s at pH 7.4 and 8.2–38.9 × 10^−6^ cm/s at pH 5.5. AC-M110 remained near the lower threshold at pH 5.5 (3.4–4.5 × 10^−6^ cm/s), while AC-R008 showed consistently higher permeability across all conditions, reaching 92 × 10^−6^ cm/s at pH 7.4 (Table 1).

Metabolic stability was analyzed in human, rat, and mouse liver microsomes, with verapamil as a control (Figure 3F; Table 1). Both AC-M110 and AC-R008 were rapidly metabolized, showing short half-life and high intrinsic clearance compared to verapamil. AC-M110 was the least stable. 1n mouse microsomes, less than 1% of the parent compound remained after 15 min, with almost complete degradation by 60 min. Similar patterns were observed in rat and human microsomes, with half-lives of 2, 9, and 6 min, respectively. Intrinsic clearance was especially high in mouse (CLint mic = 339 μL min^−1^ mg^−1^; CLint hep = 1,372 μL min^−1^ kg^−1^).

AC-R008 showed a comparable but slightly less pronounced instability. In mouse microsomes, the compound decreased rapidly, leaving only 5% after 5 min and less than 1% at 15 min. In rat and human microsomes, degradation was slower, with 55% and 47% remaining at 5 min, decreasing to about 1% at 60 min. Half-lives were 3, 10, and 7 min, respectively, and clearance values were high, particularly in the mouse (CLint mic = 501 μL min^−1^ mg^−1^; CLint hep = 2028 μL min^−1^ kg^−1^).

Verapamil, used as reference, was also rapidly metabolized but remained more stable than both test compounds, with half-lives of 19, 20, and 27 min in mouse, rat, and human, respectively (Figure 3F; Table 1).

### 3.5 The new sulfonamides reduce parasitemia at peak infection but do not alter tissue parasite load

**FIGURE 4:**
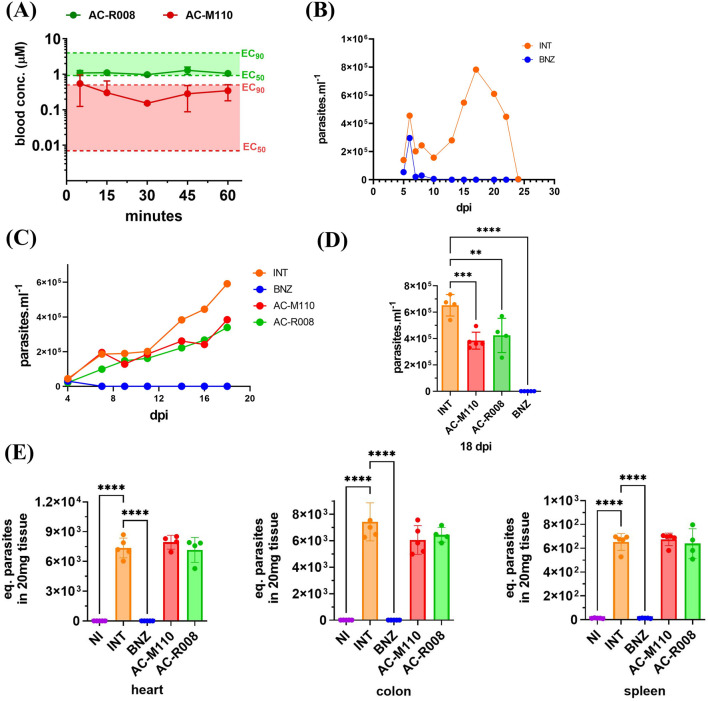
Bioavailability and *in vivo* efficacy. **(A)** Sulfonamides free plasma concentration during the first hour following oral administration of 50 mg/kg to three healthy mice (n = 3). **(B)** Animal model of acute Chagas disease using *T. cruzi*, Dm28c strain (DTU TcI) and Balb/c mice. Parasitemia was monitored over 30 days in infected and non-treated animals (INT) and infected treated with BNZ (benznidazole 50 mg/kg, SID for 14 days) treated animals. **(C)** Parasitemia curves of animals randomly assigned to groups (n = 5): INT (infected non-treated), BNZ (infected treated with benznidazole 50 mg/kg SID for 14 days), AC-M110, and AC-R008 (infected and treated with sulfonamides 150 mg/kg SID for 14 days). A group of non-infected and non-treated (NI) animals was maintained under the same conditions. **(D)** Comparison of compounds efficacy at the second parasitemia peak (18 dpi). **(E)** Quantification of *T. cruzi* DNA in heart, colon, and spleen samples from infected mice at 20 dpi. Bars represent mean ± standard deviation (SD). Statistical analysis was performed using one-way ANOVA followed by Dunnett’s multiple comparison test. Adjusted p values are indicated by asterisks (*p ≤ 0.05; **p ≤ 0.01; ***p ≤ 0.001; ****p ≤ 0.0001).

To test the *in vivo* bioavailability of the newly synthesized sulfonamides, compounds AC-M110 and AC-R008 were administered orally to mice at a single dose of 50 mg/kg, and their concentration in plasma was assessed at 5, 15, 30, 45, and 60 min. The results show that both lead candidates exhibited relatively stable plasma concentrations, between EC_50_ and EC_90_, over 1 hour (Figure 4A).

Before testing AC-M110 and AC-R008, the pilot experiment revealed prolongated parasitemia, characterized by two distinct peaks and standing for nearly 25 days. After that period, parasitemia decreased, without animal death and undetectable parasite load in heart, colon, and spleen, as assessed by qPCR at day 30 (Figure 4B).

**FIGURE 5:**
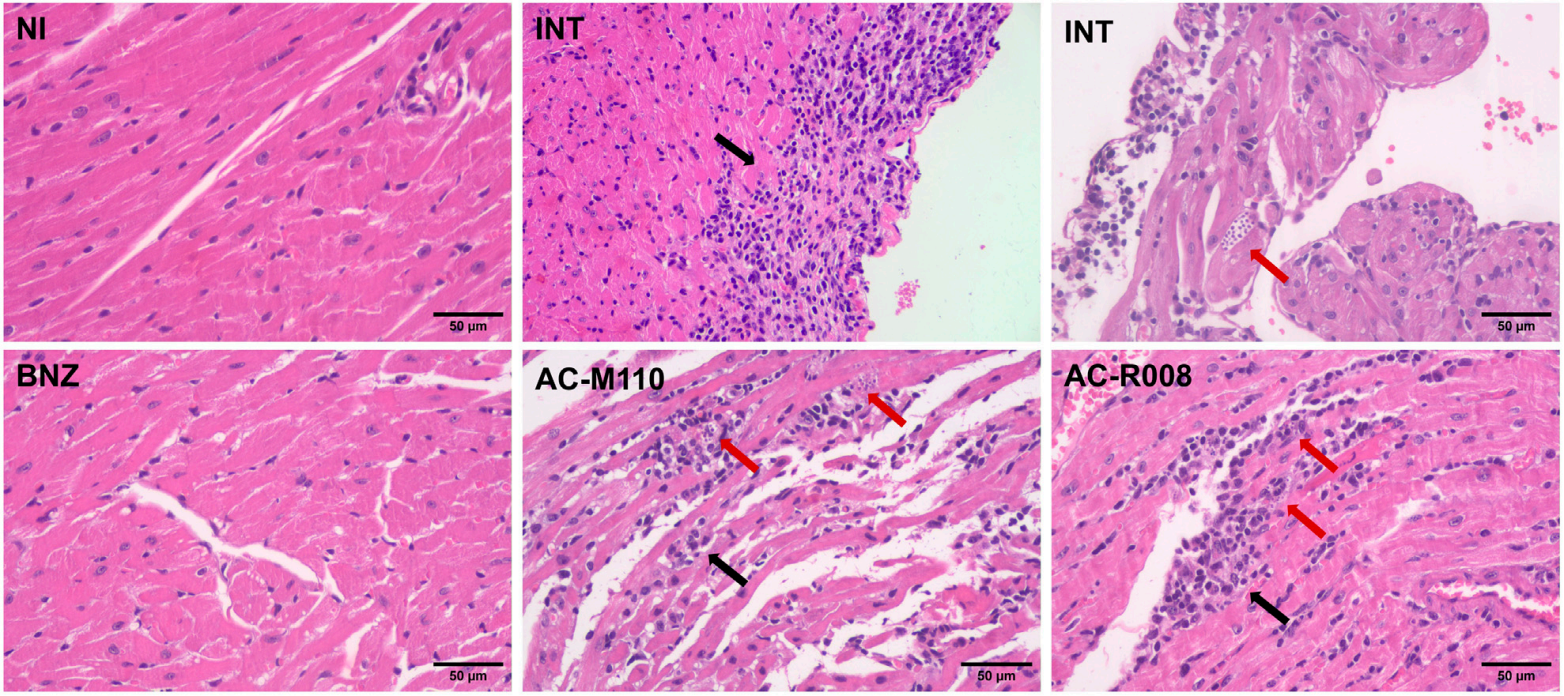
Histopathological analysis of cardiac tissue from mice infected with *Trypanosoma cruzi.* Representative photomicrographs of heart sections stained with hematoxylin and eosin (H&E), obtained at 20 days post-infection (dpi). Groups include: NI (non-infected and non-treated), INT (infected untreated), BNZ (infected treated with benznidazole), and Sulfonamides (infected treated with AC-M110 and AC-R008). Black arrows indicate confluent inflammatory foci observed in the myocardium, while red arrows highlight nests of intracellular amastigotes. Images were captured at ×200 magnification using a bright-field microscope.

A tolerability assay was conducted before the efficacy study, and no changes were observed in the evaluated animals (Supplementary Table S1). *In vivo* efficacy, assays show that AC-R008 and AC-M110 significantly reduced the second peak of parasitemia by approximately 50%, compared to infected/non-treated animals (Figures 4C,D). However, both compounds failed to reduce parasite load in the evaluated tissues: heart, colon, and spleen (Figure 4E). Histopathologycal analysis revealed confluent mixed inflammatory foci and the presence of amastigote nests in the myocardium (Figure 5). Treatment with BNZ reduced tissue inflammation and eliminated detectable amastigote nests. In contrast, the remaining groups displayed extensive chronic active inflammation and occasional amastigote nests, indicating persistent infection and active myocarditis (Table 2).

**TABLE 2:** Quantitative histopathology assessment: frequency of inflammatory foci (chronic active inflammation) and amastigote nests.

Groups	No. of inflammatory foci/animal (Mean + SD)	No. of amastigote nests/animal (Mean + SD)
NI	0	0
INT	61.4 ± 15	0.4 ± 0.5
BNZ	0.6 ± 0.5	0
AC-M110	51.2 ± 20	0.6 ± 0.5
AC-R008	55.2 ± 11	0.2 ± 0.4

NI: non-infected and non-treated; INT: infected untreated; BNZ: infected treated with benznidazole; AC-M110, and AC-R008: infected and treated with sulfonamides.

## 4 Discussion

In a previous study, we performed a screening of the Chagas Box collection against TcME (cytosolic isoform) and identified TCMDC-143108 as a potent inhibitor of this enzyme (IC_50_ = 0.08 µM) (Mercaldi et al., 2021). Building on these results, we planned structural modifications at strategic positions of the sulfonamide scaffold, enabling the generation of a small series of derivatives for *hit-to-lead* optimization. The systematic modifications introduced in TCMDC-143108 demonstrated structural features necessary for activity against TcME and the intracellular forms of *T. cruzi*. The design of this new series of sulfonamide analogs was supported by previously solved TcME crystal structures in complex with the parent hit compound (Mercaldi et al., 2021). This data revealed the allosteric site at the dimer interface and the key residues involved in ligand binding. We investigated structural modifications on one side of the molecule by replacing the methoxy group with isopropoxy, and on the opposing side, by exploring variations regarding the position of the arylamide group. The replacement of the methoxy by an isopropoxy group improved antiparasitic activity of the new molecule (AC-M109), but also increased the lipophilicity, raising concerns about solubility and bioavailability. To overcome this issue, the 3-5-difluorophenyl was replaced by a pyrazine group, effectively reducing lipophilicity while maintaining strong antiparasitic activity (AC-M110). Although such modification decreased TcME inhibition, suggesting a trade-off between target inhibition and antiparasitic activity. The synthesis of AC-R008, which restored the methoxy substituent while retaining the pyrazine group, represented an effective strategy to balance these opposing requirements, preserving both enzymatic inhibition and favorable lipophilicity, making it a promising candidate for further evaluation. The other compounds, AC-R009, AC-R010, and AC-R001, as well as the intermediates, were not active against TcME and didn´t affect the parasite. These results indicate the requirement of arylamides in the meta orientation for biological activity. Based on the antiparasitic activity (EC_50_ < 1 µM), superior to benznidazole, and favorable physicochemical properties (cLogP <3), compounds AC-M109, AC-M110, and AC-R008 progressed to subsequent assays.

The evaluation of *in vitro* ADME properties provided important insights for new drug candidates. Drug solubility and stability are critical for *in vivo* performance, as they directly influence pharmacokinetic parameters. Compounds with low solubility and stability tend to exhibit limited bioavailability (Di et al., 2012). In this study, the compounds AC-M110 and AC-R008 showed favorable solubility and stability above 60% compared to the reference drug (Alprenolol). Notably, a subtle increase in solubility and stability at alkaline pH suggests greater ionization of these compounds in intestinal environments, particularly in the ileum and colon (pH 7–8) (Delbaere et al., 2023), potentially facilitating dissolution and absorption in these regions. Although this pH-dependent increase was also observed for compound AC-M109, it did not reach the minimum performance required to proceed to subsequent experiments.

A crucial characteristic for oral administration of a drug candidate is the ability to permeate cellular barriers (Lou et al., 2025). Permeability studies indicated that AC-M110 and AC-R008 possess properties favorable for oral absorption, meeting high-permeability criteria in both Caco-2 and PAMPA models. The higher LogD_7.4_ of AC-M110 compared to AC-R008 can be attributed to the substitution of the methoxy group with isopropoxy, the only chemical difference between the molecules. Despite reducing hydrophobicity, the isopropoxy group appears to facilitate cytoplasmic entry of AC-M110, enhancing access to intracellular parasite forms.

Overall, the Caco-2 and PAMPA results classify AC-M110 and AC-R008 as highly permeable compounds, although with notable differences: AC-M110 exhibits pH-dependent behavior associated with its ionization profile, whereas AC-R008 shows superior passive permeability compared to both AC-M110 and the reference verapamil (Sun et al., 2017; Kansy et al., 2004; Chen et al., 2008; Avdeef et al., 2007; Kansy et al., 1998).

The microsomal stability assays returned short half-lives and elevated intrinsic clearance values, particularly in mouse microsomes. AC-R008 showed slightly better stability in rat and human microsomes at early time points when compared to AC-M110. However, both compounds underwent almost complete degradation within 60 min. This metabolic instability points to extensive hepatic metabolism, likely mediated by phase I enzymes, and suggests a reduced systemic exposure (Almazroo et al., 2017).

Despite these challenges, *in vivo* bioavailability demonstrated that both compounds, AC-M110 and AC-R008, achieved stable plasma concentrations over 1 h, but not surpassing the EC_90_. The pharmacokinetic profile, maintaining plasma levels between EC_50_ and EC_90_ for approximately 1 hour, suggests a short-lived exposure likely associated with a Cmax-driven pharmacodynamic effect. The transient parasitemia reduction observed *in vivo* is consistent with this limited exposure period and the high intrinsic clearance demonstrated in microsomal assays. Therefore, it is reasonable to infer that prolonged systemic exposure would be required to sustain antiparasitic activity and achieve tissue-stage efficacy. As reported by de Oliveira et al. (2025), integrating the high potency of drug candidates with appropriate pharmacokinetic properties is a significant challenge.

Considering the knowledge acquired in the present study, future efforts should prioritize the identification and management of compounds’ metabolic soft spots by chemical structure optimization. In addition, the investigation of alternative controlled-release formulations will aim to ensure that the drug candidate remains in the bloodstream for a longer period. With these issues solved, the pharmacokinetic profile will be reassessed, and a new *in vivo* efficacy assay will be repeated.

## 5 Conclusion

The evaluation of TMCD-143108 derivatives demonstrated that strategic chemical modifications upon the sulfonamide scaffold can enhance *T. cruzi in vitro* activity and improve physicochemical properties without compromising target engagement. Among them, AC-M110 and AC-R008 displayed partial parasitemia suppression without demonstrated curative potential. This limited outcome is consistent with their pronounced metabolic instability and unfavorable pharmacokinetic profiles. Future efforts will focus on structural optimization and formulation strategies aimed at improving systemic exposure and sustaining therapeutic drug levels to achieve more effective anti-*T. cruzi* activity.

## Data Availability

The original contributions presented in the study are included in the article/Supplementary Material, further inquiries can be directed to the corresponding authors.

## References

[B1] Almazroo O. A. Miah M. K. Venkataramanan R. (2017). Drug metabolism in the liver. Clin. Liver Dis. 21 (1), 1–20. 10.1016/j.cld.2016.08.001 27842765

[B2] Avdeef A. Bendels S. Di L. Faller B. Kansy M. Sugano K. (2007). PAMPA--critical factors for better predictions of absorption. J. Pharm. Sci. 96 (11), 2893–2909. 10.1002/jps.21068 17803196

[B3] Bivona A. E. Alberti A. S. Cerny N. Trinitario S. N. Malchiodi E. L. (2020). Chagas disease vaccine design: the search for an efficient Trypanosoma cruzi immune-mediated control. Biochim. Biophys. Acta Mol. Basis Dis. 1866 (5), 165658. 10.1016/j.bbadis.2019.165658 31904415

[B4] Chatelain E. Scandale I. (2020). Animal models of chagas disease and their translational value to drug development. Expert Opin. Drug Discov. 15 (12), 1381–1402. 10.1080/17460441.2020.1806233 32812830

[B5] Chen X. Murawski A. Patel K. Crespi C. L. Balimane P. V. (2008). A novel design of artificial membrane for improving the PAMPA model. Pharm. Res. 25, 1511–1520. 10.1007/s11095-007-9517-8 18185985

[B6] Cordeiro A. T. (2019). NADPH producing enzymes as promising drug targets for chagas disease. Curr. Med. Chem. 26 (36), 6564–6571. 10.2174/0929867325666181009152844 30306853

[B7] de Oliveira R. G. Cruz L. R. Dessoy M. A. Koovits P. J. Dos Santos D. A. de Oliveira L. F. N. (2025). Discovery and early optimization of 1H-Indole-2-carboxamides with anti-trypanosoma cruzi activity. J. Med. Chem. 68 (7), 7313–7340. 10.1021/acs.jmedchem.4c02942 40163677 PMC11998015

[B8] Delbaere K. Roegiers I. Bron A. Durif C. Van de Wiele T. Blanquet-Diot S. (2023). The small intestine: dining table of host-microbiota meetings. FEMS Microbiol. Rev. 47 (3), fuad022. 10.1093/femsre/fuad022 37193669 PMC10246847

[B9] Di L. Fish P. V. Mano T. (2012). Bridging solubility between drug discovery and development. Drug Discov. Today 17 (9-10), 486–495. 10.1016/j.drudis.2011.11.007 22138563

[B10] Epa U. (1996). “Product properties test guideline,” in OPPTS 830.7550 partition coefficient (n octanol/H2O), shake flask method.

[B11] Fredo Naciuk F. Do Nascimento Faria J. Goncąlves Eufrásio A. Torres Cordeiro A. Bruder M. (2020). Development of selective steroid inhibitors for the Glucose-6-Phosphate dehydrogenase from Trypanosoma Cruzi. ACS Med. Chem. Lett. 11 (6), 1250–1256. 10.1021/acsmedchemlett.0c00106 32551008 PMC7294730

[B12] Gad S. C. Spainhour C. B. Shoemake C. Pallman D. R. Stricker-Krongrad A. Downing P. A. (2016). Tolerable levels of nonclinical vehicles and formulations used in studies by multiple routes in multiple species with notes on methods to improve utility. Int. J. Toxicol. 35 (2), 95–178. 10.1177/1091581815622442 26755718

[B13] Kansy M. Senner F. Gubernator K. (1998). Physicochemical high throughput screening: parallel artificial membrane permeation assay in the description of passive absorption processes. J. Med. Chem. 41 (7), 1007–1010. 10.1021/jm970530e 9544199

[B14] Kansy M. Avdeef A. Fischer H. (2004). Advances in screening for membrane permeability: high resolution PAMPA for medicinal chemists. Drug Discov. Today Technol. 1, 349–355. 10.1016/j.ddtec.2004.11.013 24981614

[B15] Kerns E. H. Di L. (2008). Drug-like properties: concepts, structure design and methods. Elsevier.

[B16] Khare S. Roach S. L. Barnes S. W. Hoepfner D. Walker J. R. Chatterjee A. K. (2015). Utilizing chemical genomics to identify cytochrome b as a novel drug target for chagas disease. PLoS Pathog. 11 (7), e1005058. 10.1371/journal.ppat.1005058 26186534 PMC4506092

[B17] Khare S. Nagle A. S. Biggart A. Lai Y. H. Liang F. Davis L. C. (2016). Proteasome inhibition for treatment of leishmaniasis, chagas disease and sleeping sickness. Nature 537 (7619), 229–233. 10.1038/nature19339 27501246 PMC5161665

[B18] Lou H. Feng M. Al-Tamimi Z. Kuczera K. Hageman M. J. (2025). Predicting distribution coefficients (LogD) of cyclic peptides using molecular dynamics simulations. Pharm. Res. 42 (4), 613–622. 10.1007/s11095-025-03850-2 40140127

[B19] Mercaldi G. F. Eufrásio A. G. Ranzani A. T. do Nascimento Faria J. Mota S. G. R. Fagundes M. (2021). *Trypanosoma cruzi* malic enzyme is the target for sulfonamide hits from the GSK chagas box. ACS Infect. Dis. 7 (8), 2455–2471. 10.1021/acsinfecdis.1c00231 34279922

[B20] Murta S. M. F. Lemos Santana P. A. Jacques Dit Lapierre T. J. W. Penteado A. B. El Hajje M. Navarro Vinha T. C. (2024). New drug discovery strategies for the treatment of benznidazole-resistance in *Trypanosoma cruzi*, the causative agent of chagas disease. Expert Opin. Drug Discov. 19 (6), 741–753. 10.1080/17460441.2024.2349155 38715393

[B21] Obach R. S. (1999). Prediction of human clearance of twenty-nine drugs from hepatic microsomal intrinsic clearance data: an examination of *in vitro* half-life approach and nonspecific binding to microsomes. Drug Metab. Dispos. 27 (11), 1350–1359. 10.1016/s0090-9556(24)14938-0 10534321

[B22] Ogilvie B. W. Usuki E. Yerino P. Parkinson A. (2008). *In vitro* approaches for studying the inhibition of drug-metabolizing enzymes and identifying the drug-metabolizing enzymes responsible for the metabolism of drugs (reaction phenotyping) with emphasis on cytochrome P450. Drug-drug Interactions, 231–358. 10.1201/9780429131967-7

[B23] Peña I. Pilar Manzano M. Cantizani J. Kessler A. Alonso-Padilla J. Bardera A. I. (2015). New compound sets identified from high throughput phenotypic screening against three kinetoplastid parasites: an open resource. Sci. Rep. 5, 8771. 10.1038/srep08771 25740547 PMC4350103

[B24] Ranzani A. T. Nowicki C. Wilkinson S. R. Cordeiro A. T. (2017). Identification of specific inhibitors of *Trypanosoma cruzi* malic enzyme isoforms by target-based HTS. SLAS Discov. 22 (9), 1150–1161. 10.1177/2472555217706649 28459632

[B25] Rial M. S. Reigada C. Prado N. Bua J. Esteva M. Pereira C. A. (2023). Effectiveness of the repurposed drug isotretinoin in an experimental murine model of chagas disease. Acta Trop. 242, 106920. 10.1016/j.actatropica.2023.106920 37028584

[B26] Soeiro M. N. C. Sales-Junior P. A. Pereira V. R. A. Vannier-Santos M. A. Murta S. M. F. de Sousa A. S. (2024). Drug screening and development cascade for chagas disease: an update of *in vitro* and *in vivo* experimental models. Memórias Do Inst. Oswaldo Cruz 119, e240057. 10.1590/0074-02760240057 38958341 PMC11218046

[B27] Sun H. Nguyen K. Kerns E. Yan Z. Yu K. R. Shah P. (2017). Highly predictive and interpretable models for PAMPA permeability. Bioorg. Med. Chem. 25, 1266–1276. 10.1016/j.bmc.2016.12.049 28082071 PMC5291813

[B28] World Health Organization (2024). Chagas disease (American trypanosomiasis). Available online at: https://www.who.int/news-room/fact-sheets/detail/chagas-disease-(american-trypanosomiasis).

