# Supplementary material for: Efficacy of sulfonamides targeting malic enzyme in an animal model of Chagas disease
Source: Front Pharmacol. 2025 Nov 28;16:1709223. doi: 10.3389/fphar.2025.1709223 (PMC12698583; doi:10.3389/fphar.2025.1709223)
Supplement: Supplementary file 1 [file Supplementaryfile1.docx]

**Supplementary Material**

**Efficacy of sulfonamides targeting malic enzyme in an animal model of Chagas disease**

Thaís Cristina Ferreira dos Santos^1,†*^, Ramon Borges da Silva^1,†^, Irene Layane de Sousa^1^, Fabrício Fredo Naciuk^1^ Letícia Marchese^1^, Amanda Gonçalves Eufrásio^1,2^, Angel Eduardo Lobo-Rojas^1^, Renan Marcel Giampauli^1^, Valéria Barbosa de Souza^3^, André Almeida Schenka^3^, Marjorie Bruder^1^, Silvana Aparecida Rocco^1^, Artur Torres Cordeiro^1,*^

^1^Brazilian Biosciences National Laboratory, Brazilian Center for Research in Energy and Materials, Campinas, São Paulo, Brazil.

^2^Faculty of Pharmaceutical Sciences, University of Campinas, São Paulo, Brazil.

^3^Department of Pharmacology, Faculty of Medical Sciences, University of Campinas, São Paulo, Brazil.

*Corresponding authors: Thaís Cristina Ferreira dos Santos, [thais.santos@lnbio.cnpem.br](mailto:thais.santos@lnbio.cnpem.br), and Artur Torres Cordeiro, [artur.cordeiro@lnbio.cnpem.br](mailto:artur.cordeiro@lnbio.cnpem.br), Brazilian Biosciences National Laboratory – LNBio, Brazilian Center for Research in Energy and Materials – CNPEM, 10,000 Giuseppe Maximo Scolfaro St, Campinas, SP, Brazil 13083-100.

**Summary**

NMR spectra of sulfonamides..................................................................................................3

UV-HPLC purity profile of selected compounds ...................................................................20

Kinetic solubility and chemical stability.................................................................................22

Stability in HBSS buffer.........................................................................................................24

Tolerability study for the Hippocratic Screening....................................................................25

References................................................................................................................................26

**NMR spectra of Sulfonamides**


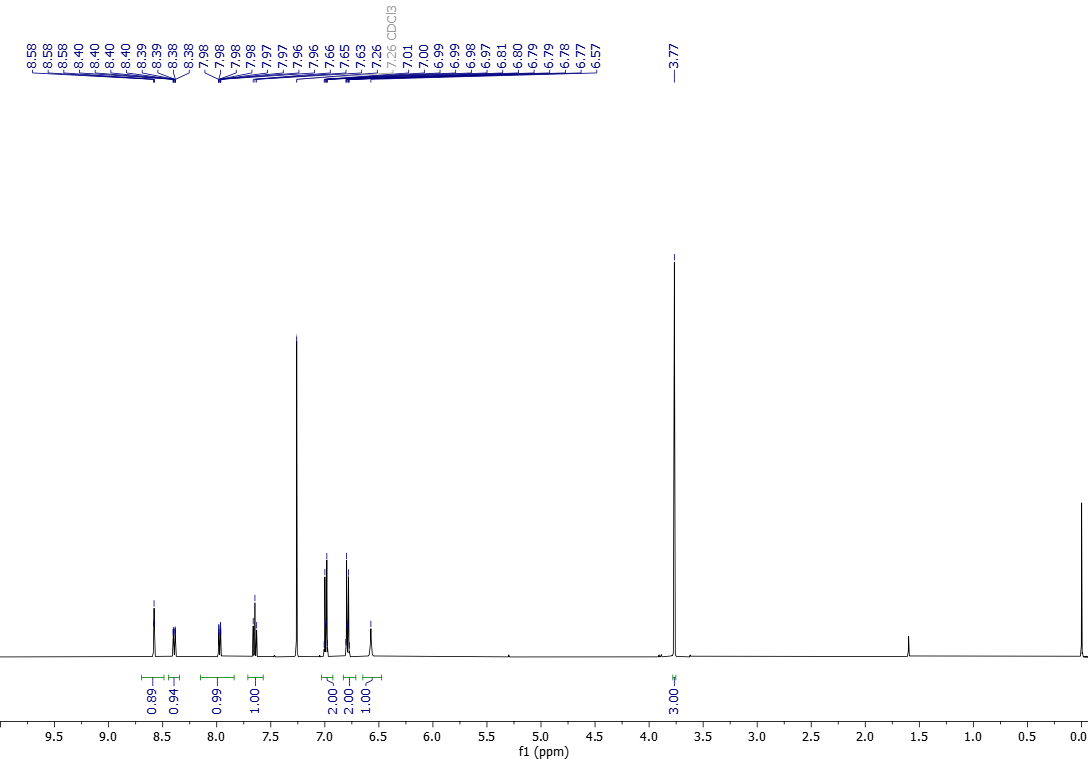
^1^H NMR: *N*-(4-methoxyphenyl)-3-nitrobenzenesulfonamide (**5**), (500 MHz, CDCl_3_).


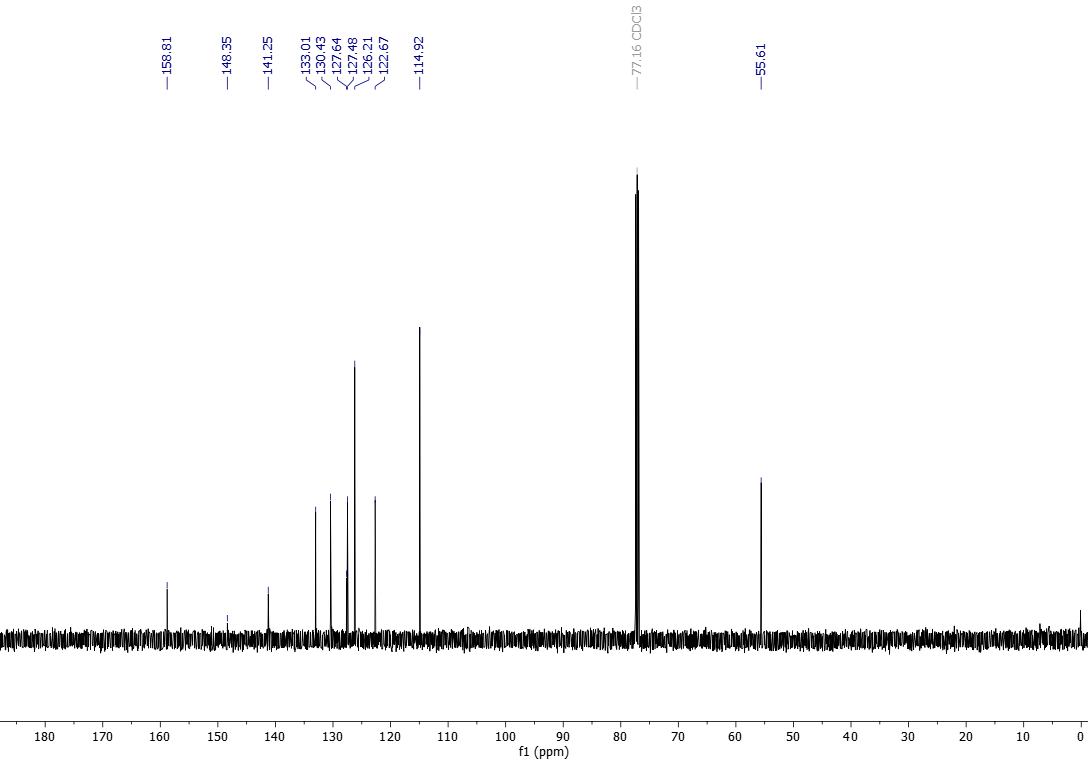
^13^C NMR: *N*-(4-methoxyphenyl)-3-nitrobenzenesulfonamide (**5**), (126 MHz, CDCl_3_).


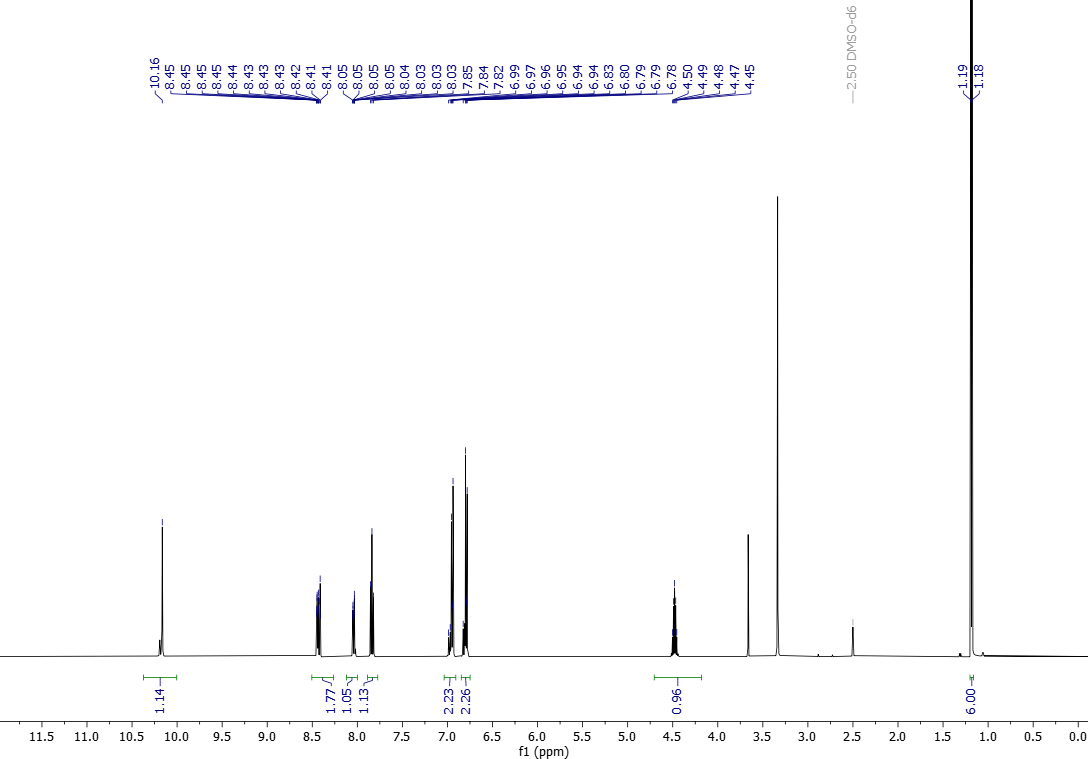


^1^H NMR: *N*-(4-isopropoxyphenyl)-3-nitrobenzenesulfonamide (**6**), (500 MHz, DMSO-_d6_).


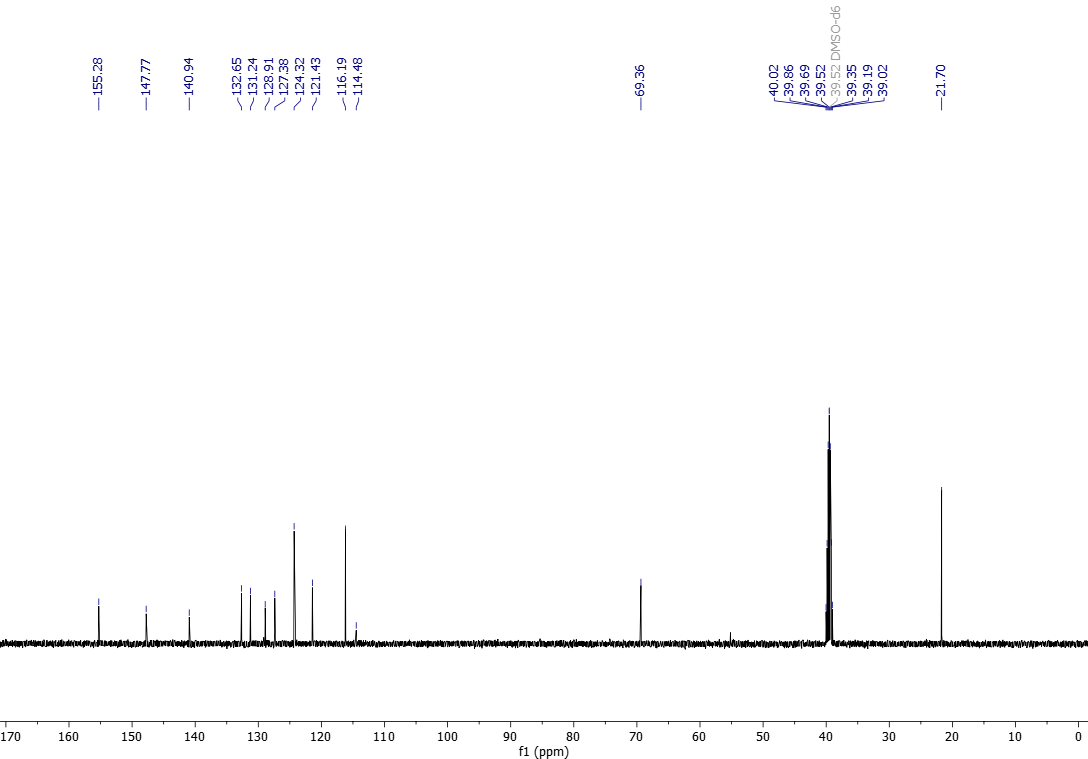
^13^C NMR: *N*-(4-isopropoxyphenyl)-3-nitrobenzenesulfonamide (**6**), (126 MHz, DMSO-_d6_).

^1^H NMR: *N*-(4-methoxyphenyl)-4-nitrobenzenesulfonamide (**7**), (500 MHz, DMSO-_d6_).

^13^C NMR: *N*-(4-methoxyphenyl)-4-nitrobenzenesulfonamide (**7**), (126 MHz, DMSO-_d6_).

^1^H NMR: *N*-(4-isooxyphenyl)-4-nitrobenzenesulfonamide (**8**), (500 MHz, DMSO-_d6_).

^13^C NMR: *N*-(4-isooxyphenyl)-4-nitrobenzenesulfonamide (**8**), (126 MHz, DMSO-_d6_).

^1^H NMR: 3-amino-*N*-(4-methoxyphenyl)benzenesulfonamide (**9**), (500 MHz, DMSO-_d6_).

^13^C NMR: 3-amino-*N*-(4-methoxyphenyl)benzenesulfonamide (**9**), (126 MHz, DMSO-_d6_).

^1^H NMR: 3-amino-N-(4-isopropoxyphenyl)benzenesulfonamide (**10**), (500 MHz, DMSO-_d6_).

^13^C NMR: 3-amino-N-(4-isopropoxyphenyl)benzenesulfonamide (**10**), (126 MHz, DMSO-_d6_).

^1^H NMR: 4-amino-*N*-(4-metoxyphenyl)benzenesulfonamide (**11**), (500 MHz, DMSO-_d6_).

^13^C NMR: 4-amino-*N*-(4-metoxyphenyl)benzenesulfonamide (**11**), (126 MHz, DMSO-_d6_).

^1^H NMR: 4-amino-*N*-(4-isopropoxyphenyl)benzenesulfonamide (**12**), (500 MHz, DMSO-_d6_).

^13^C NMR: 4-amino-*N*-(4-isopropoxyphenyl)benzenesulfonamide (**12**), (126 MHz, DMSO-_d6_).

^1^H NMR: *N*-(3-(*N*-(4-methoxyphenyl)sulfamoyl)phenyl)pyrazine-2-carboxamide (**13**), (500 MHz, DMSO-_d6_).

^13^C NMR: *N*-(3-(*N*-(4-methoxyphenyl)sulfamoyl)phenyl)pyrazine-2-carboxamide (**13**), (126 MHz, DMSO-_d6_).

^1^H NMR: *N*-(3-(*N*-(4-isopropoxyphenyl)sulfamoyl)phenyl)pyrazine-2-carboxamide (**14**), (500 MHz, DMSO-_d6_).

^13^C NMR: *N*-(3-(*N*-(4-methoxyphenyl)sulfamoyl)phenyl)pyrazine-2-carboxamide (**14**), (126 MHz, DMSO-_d6_).

^1^H NMR: *N*-(4-(*N*-(4-methoxyphenyl)sulfamoyl)phenyl)pyrazine-2-carboxamide (**15**), (500 MHz, DMSO-_d6_).

^13^C NMR: *N*-(4-(*N*-(4-methoxyphenyl)sulfamoyl)phenyl)pyrazine-2-carboxamide (**15**), (126 MHz, DMSO-_d6_).

^1^H NMR: *N*-(4-(*N*-(4-isopropoxyphenyl)sulfamoyl)phenyl)pyrazine-2-carboxamide (**16**), (500 MHz, DMSO-_d6_).

 ^13^C NMR: *N*-(4-(*N*-(4-isopropoxyphenyl)sulfamoyl)phenyl)pyrazine-2-carboxamide (**16**), (126 MHz, DMSO-_d6_).

^1^H NMR: 3,5-difluoro-*N*-(4-(*N*-(4-methoxyphenyl)sulfamoyl)phenyl)benzamide (**17**), (500 MHz, DMSO-_d6_).

^13^C NMR: 3,5-difluoro-*N*-(4-(*N*-(4-methoxyphenyl)sulfamoyl)phenyl)benzamide (**17**), (126 MHz, DMSO-_d6_).

^1^H NMR: 3,5-difluoro-*N*-(3-(*N*-(4-isopropoxyphenyl)sulfamoyl)phenyl)benzamid*e* (**18**), (500 MHz, DMSO-_d6_).

^13^C NMR: 3,5-difluoro-*N*-(3-(*N*-(4-isopropoxyphenyl)sulfamoyl)phenyl)benzamid*e* (**18**), (126 MHz, DMSO-_d6_).

**UV-HPLC purity profile of selected compounds**

**AC-M110**


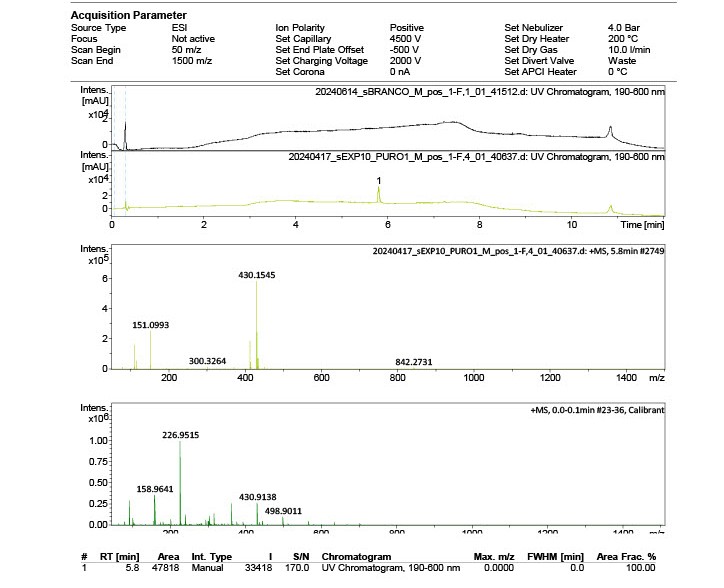


**AC-R008**


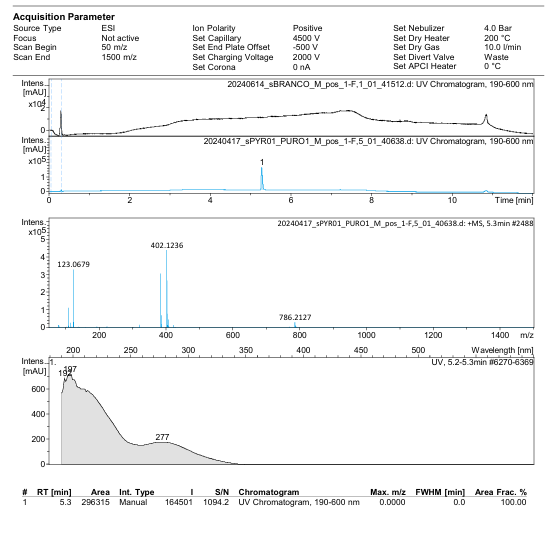


**Kinetic solubility and chemical stability**

Experimental protocol

The compounds AC-M110 and AC-R008 were prepared at a concentration of 5 µM in 0.4% DMSO and evaluated at pH 1.7 (Clark-Lubs buffer, 0.2 M), pH 7.4 (sodium phosphate buffer, 0.1 M), and pH 8.9 (Tris buffer adjusted with 2 M hydrochloric acid). These conditions simulate stomach acidity, plasma neutrality, and intestinal basicity. For the solubility assay, the solutions were maintained at 25°C (Thermal Mixer, Eppendorf), and samples (200 μL) were collected at 0 and 1.5 hours. For the chemical stability experiment, the compounds were evaluated under the same conditions with an additional incubation at 37°C for a period of 24 hours. The analysis was performed using an analytical method developed and validated by high-performance liquid chromatography with UV detection (HPLC-UV). Compounds were quantified in the different pH media by comparing the areas where the substances were completely soluble with those in the reaction media. The experiments were performed in triplicate.


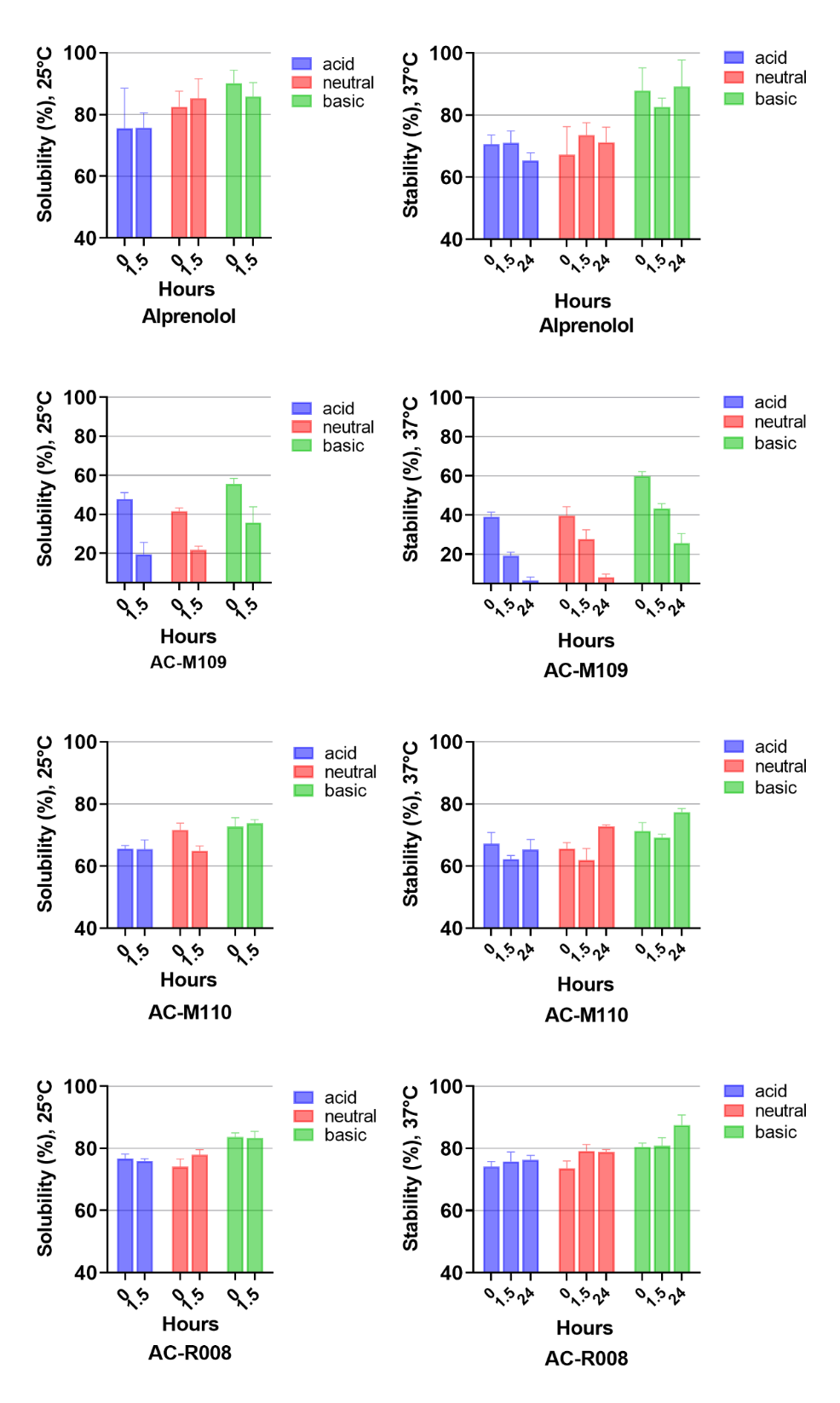


**Figure S1. Kinetic solubility and chemical stability of AC-M110 and AC-R008 under different pH conditions.** Compounds were evaluated at pH 1.7, 7.4, and 8.9 to simulate gastric, plasma, and intestinal environments, respectively. Solubility was determined at 0 and, 1.5 hours at 25°C, while chemical stability was assessed at 0, 1.5 and, 24 hours at 37°C. Quantification was performed by HPLC-UV. Data represents the mean ± SD of the triplicate.

**Stability in HBSS buffer**

Experimental protocol

The compounds were evaluated for chemical stability in Hanks’ Balanced Salt Solution (HBSS) over a 2-hour incubation period at 37°C. Stock solutions prepared in DMSO were diluted in HBSS to the desired test concentration. Samples were collected at time zero and after 2 hours of incubation. The remaining concentration of each compound was quantified by HPLC-UV, with stability assessed by comparing the percentage of compound remaining after 2 hours relative to the initial concentration.


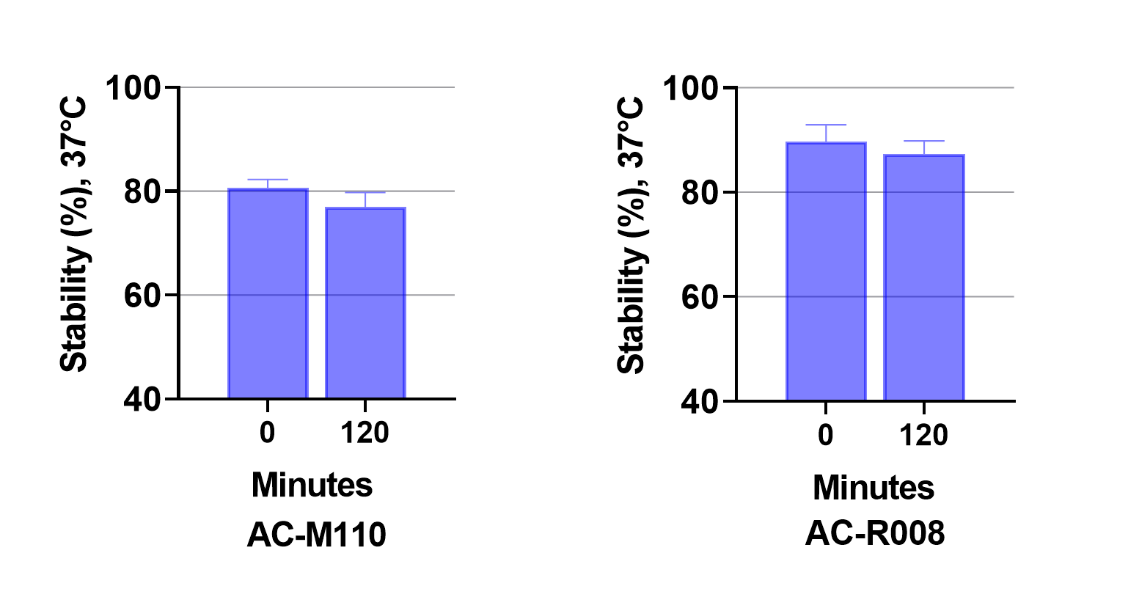


**Figure S2. Chemical stability of AC-M110 and AC-R008 in HBSS buffer prior to the permeability assay.** To ensure compound stability under the conditions used for the permeability assay, AC-M110 and AC-R008 were incubated in HBSS buffer at 37°C for 2 hours. Compound integrity was assessed by HPLC-UV. Data represents the mean ± SD of the triplicate. The data confirm that both compounds remained chemically stable during the incubation period, supporting their use in subsequent permeability testing.

**Tolerability study for the Hippocratic Screening**

A tolerability study of drug candidates was conducted using the Hippocratic screening protocol, focusing on the assessment of effects on the central nervous system (CNS) and autonomic nervous system (ANS). Each group containing 3 animals received a 150 mg/kg dose of the compounds via oral (PO) administration (gavage). During the experiment, various behavioral and physiological parameters were monitored at regular intervals, ranging from 0 to 24 hours after administration of the substance.

**Table 1S**. Acute toxicity parameters were monitored up to 24 hours for compounds AC-M110 and AC-R008.

| **CNS/ANS**      **Time** | 0-15 min | 15 min | 30 min | 60 min | 2h | 3h | 4h | 24h |
| --- | --- | --- | --- | --- | --- | --- | --- | --- |
| Convulsions | 0A | 0A | 0A | 0A | 0A | 0A | 0A | 0A |
| Pedaling | 0A | 0A | 0A | 0A | 0A | 0A | 0A | 0A |
| Salivation | 0A | 0A | 0A | 0A | 0A | 0A | 0A | 0A |
| Tremors | 0A | 0A | 0A | 0A | 0A | 0A | 0A | 0A |
| Vocalization | 0A | 0A | 0A | 0A | 0A | 0A | 0A | 0A |
| Limb abduction | 0A | 0A | 0A | 0A | 0A | 0A | 0A | 0A |
| Ataxia | 0A | 0A | 0A | 0A | 0A | 0A | 0A | 0A |
| Hypnosis | 0A | 0A | 0A | 0A | 0A | 0A | 0A | 0A |
| Ptosis | 0A | 0A | 0A | 0A | 0A | 0A | 0A | 0A |
| Sedation | 0A | 0A | 0A | 0A | 0A | 0A | 0A | 0A |
| Urination | 0A | 0A | 0A | 0A | 0A | 0A | 0A | 0A |
| Piloerection | 0A | 0A | 0A | 0A | 0A | 0A | 0A | 0A |
| Tachypnea | 0A | 0A | 0A | 0A | 0A | 0A | 0A | 0A |
| Muscle tone | 0A | 0A | 0A | 0A | 0A | 0A | 0A | 0A |

**Classification of effects:** (0) No effect, (1) Mild effect, (2) Moderate effect, (3) Severe effect. **Number of affected animals:** 0/3 (A), 2/3 (B), 3/3 (C) Adapted Malone (1983).

Regarding toxicity, during the 24-hour observation period, no animals exhibited apparent clinical signs, behavioral changes, or adverse effects such as convulsions, lethargy, postural alterations, motor difficulties, or signs of acute distress, suggesting a favorable initial safety profile under the experimental conditions.

**References**

Malone MH. The pharmacological evaluation of natural products--general and specific approaches to screening ethnopharmaceuticals. J Ethnopharmacol. 1983 Aug;8(2):127-47. doi: 10.1016/0378-8741(83)90050-8.
